# Pan-Cancer Analysis Reveals SOX2 as a Promising Prognostic and Immunotherapeutic Biomarker Across Various Cancer Types, Including Pancreatic Cancer

**DOI:** 10.7150/jca.88397

**Published:** 2024-01-01

**Authors:** Yuntao Ding, Huizhi Wang, Qiaowei Wang, Han Jiang, Zhangzuo Li, Zhengyue Yu, Qi Wang, Min Xu

**Affiliations:** 1 Department of Gastroenterology, Affiliated Hospital of Jiangsu University, Jiangsu University, Zhenjiang, China.; 2 Hematological Disease Institute of Jiangsu University, Affiliated Hospital of Jiangsu University, Jiangsu University, Zhenjiang, 212001, China.

**Keywords:** pan-cancer analysis, SOX2, pancreatic cancer, prognostic biomarker, molecular biomarker, immune infiltration

## Abstract

SOX2 is associated with the initiation, growth, and progression of various tumors and is related to stem cells. However, further studies of SOX2 in a pan-cancer context are warranted. In this study, we obtained pan-cancer and clinical data from TCGA, GTEx, STRING, and TISIDB databases and we analyzed the relationship between SOX2 expression levels and changes in gene diagnostics and survival prognosis. Additionally, we compared the expression levels of SOX2 in pancreatic cancer and healthy pancreatic tissues using Wilcoxon's rank-sum test. Functional enrichment analysis was conducted to identify potential signaling pathways and biological functions. To determine the prognostic value, we used the area under the curve (AUC) and Cox regression analysis. We further developed nomograms to predict overall survival at 1, 6, and 12 months after cancer diagnosis. Moreover, we assessed immune cell infiltration using single-sample gene set enrichment analysis. The methylation status of SOX2 was analyzed using the UALCAN and MethSurv databases. Furthermore, we verified the differential expression of SOX2 in pancreatic cancer cell lines by western blotting and quantitative polymerase chain reaction. We also confirmed the effect of SOX2 on the invasion and migration of pancreatic cancer cells using transwell and scratch assays. The biological effects were confirmed using a clone-formation assay. Our findings suggest that SOX2 is highly expressed in various tumor tissues and has potential clinical significance. It can be used as a new biomarker for pancreatic adenocarcinoma and plays a crucial role in immune infiltration.

## Introduction

SOX2 is a stemness-associated gene that affects the proliferation, migration, and invasion of various tumors 1. In colorectal cancer, SOX2 has been shown to work with TM4SF1 to maintain cancer cell stemness and epithelial-mesenchymal transition (EMT) 2, and thus, may be a new potential biomarker 3. In lung cancer, the PCAT1/SOX2 axis promotes tumorigenesis and immunosuppression by inhibiting cGAS/STING-signaling-mediated T cell activation 4. In addition, SOX2 interacts with CDK1 to promote lung cancer cell stemness 5. SOX2 also promotes lineage plasticity and anti-androgen resistance in TP53- and RB1-deficient prostate cancer 6. Moreover, SOX2 acts with T-Nepc to facilitate neuroendocrine prostate cancer development via the LIN28b/let-7/SOX2 axis 7. In pancreatic cancer, SOX2 makes pancreatic cancer cells resistant to gemcitabine through the GLI-SOX2 axis 8, and it controls the stemness of pancreatic cancer cells through FGFR/AKT/SOX2, which is a potential therapeutic target 9. Additionally, the inhibition of SOX2 expression by mir-1181 inhibits the stemness of pancreatic cancer cells 10.

In hepatocellular carcinoma, SOX family genes can be used to predict prognosis. Moreover, the promote an immune microenvironment, especially the infiltration of CD4+ T cells and macrophage immune cells 12. For example, SOX11, a member of the same family as SOX2, is a novel tumor therapeutic target because of its immune infiltration potential 11.

Previous studies have suggested that SOX2 is a potential therapeutic target. However, most of these studies have focused on the role of SOX2 in tumor stemness and a novel signaling axis, especially in pancreatic cancer, has not been studied in depth. Previous studies of immune infiltration have mainly focused on other SOX family genes, and there are few studies on SOX2in pancreatic cancer. Therefore, this topic warrants further research.

In this study, we performed a pan-cancer analysis of SOX2 and analyzed the expression levels of SOX2 in different tumor subtypes and immune cells using the TCGA, GETX, and TSIDB databases. STRING and PPI networks were used to explore the proteins associated with functional enrichment. The relationships between SOX2 expression levels and overall survival (OS), disease-specific survival (DSS), and the progression-free interval (PFI) were also explored. A receiver operating characteristic (ROC) curve was used to determine the predictive potential and it showed that SOX2 is a new potential target for the treatment of pancreatic cancer. A box plot, area under the curve (AUC), risk score, prognostic calibration, prognostic nomogram, and Cox regression analysis were used to validate the clinical potential of SOX2. In addition, we explored genes that are co-expressed with SOX2 in pancreatic cancer, performed Kyoto Encyclopedia of Genes and Genomes (KEGG) and Gene Ontology (GO) enrichment analysis, and verified the methylation-related indicators of SOX2. Finally, we performed a bioinformatics study of SOX2 immune infiltration in pancreatic cancer, and the results showed that high levels of SOX2 expression were positively correlated with the infiltration of γδ T (Tgd) cells, mast cells, and dendritic cells (DCs).

T cells are divided into two major categories, αβ T cells, such as CD4+ and CD8+ cells, and Tgd cells, depending on their T-cell receptor. Human peripheral blood lymphocytes are mainly αβ T cells, and Tgd cells generally account for only 1-5% 22. Although their numbers are small, their impact cannot be underestimated, as they can mobilize almost the entire immune system by themselves 23. γδ T cells can directly kill tumor cells through their cell surface natural killer (NK) cell receptors, antibody-dependent cellular cytotoxicity effect, and secreted cytokines (IFN-γ and TNF-α). γδ T cells can also activate B, DC, αβ T, and NK cells using various mechanisms, such as acting as antigen-presenting cells to activate αβ T cells or inducing NK-mediated antitumor cytotoxicity via the 4-1BB co-stimulation pathway, which in turn leads to the indirect killing of tumors 24.

In conclusion, we identified the function of SOX2 in pancreatic cancer using pan-cancer analysis. A comprehensive evaluation was performed by analyzing functional enrichment, methylation, and immune infiltration. SOX2 was found to play an important role in pancreatic cancer, especially in immune infiltration, and is thus, a potential therapeutic target and biomarker.

## Methods and materials

### Gene expression analysis

We downloaded RNAseq data from 33 types of tumors from the STAR trial from the The Cancer Genome Atlas (TCGA) database (https://portal.gdc.cancer.gov) and extracted transcripts per million reads (TPM) data. Relevant data for normal tissues and cells were downloaded from the Genotype Tissue Expression (GTEx) database. TPM were used to standardize the level 3 fragments per kilobase of transcript per million mapped reads data using HTSeq. R software v4.2.1 was used for statistical analysis, and the ggplot2 package was used for visualization. Wilcoxon's rank-sum test was used to analyze the data of the two groups, and p < 0.05 was considered statistically significant.

### SOX2 expression in molecular subtypes and immune subtypes of cancers

TISIDB (http://cis.hku.hk/TISIDB/index.php) is a web portal for tumor and immune system interaction, integrating multiple heterogeneous data types. This database evaluated the correlation of SOX2 expression in pan-cancer with molecular or immune subtypes. In addition, we evaluated the correlation between SOX2 expression and immunomodulators in pan-cancer. The full names and abbreviations of cancers are shown in tabletS1.

### Protein-protein interaction network building and functional enrichment

An online database was used using the interactive gene search tool, STRING (version 11.5; http://string-db.org), with the following main settings. A total of 50 SOX2-binding proteins were acquired, with a minimum required interaction score (“medium confidence [0.400]”) and active interaction sources (“experiments, text mining, databases, co-expression, neighborhood, gene fusion, co-occurrence”). Cytoscape (version 3.9.1) was used to construct the protein-protein interaction (PPI) network. After ID transformation of the list of input molecules, the Cluster Profile (version 4.4.4) R package was used for enrichment analyses, including GO and KEGG analyses (adjusted p-value < 0.05, false detection rate < 0.25).

### Microsatellite instability and mutant-allele tumor heterogeneity analysis of genes

We downloaded the harmonized pan-cancer dataset from the UCSC database (https://xenabrowser.net/). ENSG00000181449 (SOX2) expression data were extracted from the TCGA Pan-Cancer data set (PANCAN, N = 10,535, G = 60,499), and the sample source was further screened as primary blood-derived cancer-peripheral blood, and primary tumor samples. Using instability score data from a previous study (Landscape of Microsatellite Instability Across 39 Cancer Types, DOI:10.1200/PO.17.00073), we integrated the microsatellite instability (MSI) and gene expression data of the samples, and further applied log2(x + 0.001) transformation to each expression value. Finally, we excluded tumors with less than three samples for a single cancer type and obtained expression data for 37 cancer types. We also calculated the mutant-allele tumor heterogeneity (MATH) value for each tumor using the inferHeterogeneity function of the R package, maftools (version 2.8.05) to assess heterogeneity. We further applied log2(x + 0.001) transformation to each expression value by integrating the TMB and gene expression data of the samples, and excluded cancers with fewer than three samples for a single cancer type, resulting in expression data for 37 cancer types.

### Gene mutation landscape

We used MuTect2 software (DOI: 10.1038/nature08822) to download all level 4 TCGA sample data for the Simple Nucleotide Variation dataset from the Genomic Data Commons portal (https://portal.gdc.cancer.gov/). We integrated the mutation data of the samples and obtained the protein domain information from the R package, maftools (version 2.2.10).

### Immune checkpoint gene profiling and immunomodulatory genes analysis

We downloaded the following harmonized pan-cancer dataset from the UCSC database (https://xenabrowser.net/) TCGA TARGET GTEx (PANCAN, N = 19,131, G = 60,499), ENSG00000181449 (SOX2). Moreover, we extracted marker gene expression data for 60 two-class immune checkpoint pathway genes (24 inhibitory and 36 stimulatory) from the literature (Immune Landscape of Cancer, DOI:10.1016/j..2018.03.023). We further screened the sample source for primary solid tumors, primary tumors, primary blood-derived cancer-bone marrow, and primary blood-derived cancer-peripheral. We further applied log2(x + 0.001) transformation to each expression value, and then calculated the Pearson's correlation coefficient between ENSG00000181449 (SOX2) and the marker genes of five immune pathways. The ENSG00000181449 (SOX2) gene and 150 markers for five classes of immune pathways (41 chemokines, 18 receptors, 21 major histocompatibility molecules, 24 immunoinhibitors, and 46 immunostimulators) were extracted. Based on the expression data for each sample, we further screened the sample sources as: primary solid tumors, primary tumors, primary blood-derived cancer-bone marrow, and primary blood-derived cancer-peripheral. We further applied log2(x + 0.001) transformation to each expression value, and then calculated the Pearson's correlation coefficient between ENSG00000181449 (SOX2) and the marker genes of the five immune pathways.

### Drug susceptibility and pathway analysis

Using the GSCAlite online database (http://bioinfo.life.hust.edu.cn/web/GSCALite/), we selected the top ten genes with expression levels correlated with SOX2 expression levels for drug sensitivity and pathway analyses.

### Survival prognosis analysis

Kaplan-Meier plots were used to assess the relationship between SOX2 expression levels and cancer prognosis (OS). Proportional hazards hypothesis testing and fitted survival regression were performed using the survival R package (version 3.3.1) and the results were visualized using the Survminer and ggplot2 (version 3.3.6) R packages. The log-rank test was used for hypothesis testing, and p < 0.05 was considered statistically significant.

### Diagnostic value analysis

ROC curves were used to evaluate the diagnostic value of SOX2 in pancreatic cancer. The pROC R package (version 1.18.0) was used to analyze the data and the results were visualized using ggplot2 (version 3.3.6). By default, the pROC package corrected the outcome order of the data at a significance level of p < 0.05.

### Clinical significance of SOX2 in pancreatic adenocarcinoma

The SOX2 expression levels in patients with different clinical characteristics are presented as box plots and tables, which were constructed using ggplot2 (version 3.3.6). AUCs, risk scores, calibrations, nomograms, and forest maps were used to further analyze the clinical significance of SOX2 in pancreatic adenocarcinoma (PAAD). AUC curves were generated by analyzing the data using the timeROC R package (version 0.4), and the results were visualized using ggplot2 (version 3.3.6). The risk score maps were visualized using the ggplot2 R package (version 3.3.6). The survival R package (version 3.3.1) was used for proportional hazard hypothesis testing and Cox regression analysis, whereas the rms R package (version 6.3-0) was used for calibration analysis and visualization. The survival package was used for proportional hazard hypothesis testing and Cox regression analysis, and the rms package was used to construct and visualize the nomogram correlation model. Forest map visualization was performed using the ggplot2 R package (version 3.3.6).

### Analysis of genes co-expressed with SOX2 in PAAD

We extracted the data for corresponding genes from the selected public databases and divided them into high- and low-expression groups. The original count matrix of the selected data was analyzed according to a standard procedure using the DESeq2 R package (version 1.36.0). Using the Pearson's correlation coefficient, we also showed correlations between SOX2 expression levels and the expression levels of the top 10 genes in the heatmap. KEGG and GO analyses were used to predict functional enrichment between the co-expressed genes and SOX2. Visualization was performed using ggplot2 (version 3.3.6).

### DNA methylation analysis

To investigate the possible mechanisms of action of SOX2 in pancreatic cancer, we examined the methylation status of the SOX2 promoters using the UALCAN database (Chandrashekar et al., 2017). Additionally, the MethSurv database, an online tool for multivariate survival analysis based on DNA methylation data, was used to evaluate the predictive value of the methylation levels of SOX2 (Modhukur et al., 2018).

### Immune infiltration analysis

The extent of immune cell infiltration was calculated in 24 immune cells. The relative value of immune cell accumulation in pancreatic cancer was assessed using a single Gene Set Enrichment Analysis probe with the GSV R package (Bindea et al., 2013). Spearman's correlation analysis was used to test the correlation between the expression levels of SOX2 and immune cell infiltration. Differences in the degree of immune cell infiltration between the high- and low-expression groups were assessed using Wilcoxon's rank-sum test.

### Single cell sequencing

Tumor Immune Single-cell Hub 2 (TISCH2)( http://tisch.comp-genomics.org/) is a scRNA-seq database focusing on tumor microenvironment (TME). TISCH2 provides detailed cell-type annotation at the single-cell level, enabling the exploration of TME across different cancer types. The heat map shows the effect of SOX2 on the location of infiltration of the corresponding immune cells.

### Cell line and cell culture

The Central Laboratory of the Affiliated Hospital of Jiangsu University and the Institute of Basic Medicine of the School of Medicine of Jiangsu University stored the pancreatic cancer cell lines, MIA-PaCa-2, PaTu8988, and PANC1. PaTu8988 and PANC1 cells were cultured in Dulbecco's modified Eagle medium (Hyclone, Beijing, China) supplemented with 10% fetal bovine serum (FBS; Gibco, Carlsbad, CA, USA) and 100 mg of penicillin at 37 °C in a humidified incubator with 5% CO2 supply. The pancreatic cancer cell line, BxPc-3, was cultured in Roswell Park Memorial Institute 1640 medium (BioSharp, Talinn, Estonia) supplemented with 10% fetal bovine serum (Gibco) and 100 mg of penicillin at 37°C.

### RNA extraction and real-time polymerase chain reaction

TRIzol reagent (Invitrogen Corporation, Carlsbad, CA, USA) was used to extract total RNA. Reverse transcription was performed using the RevertAid First Strand cDNA Synthesis Kit (Thermo Fisher Scientific, Waltham, MA, USA), according to the manufacturer's specifications. Real-time polymerase chain reaction (PCR) was performed using an iQ SYBR Premix Ex Taq Perfect Real Time kit (Bio-Rad Laboratories, Inc., Hercules, CA, USA), with a 10 µL reaction volume and SYBR as the DNA-specific fluorescent dye. Human U6 was used as the housekeeping gene. The primer pairs used to amplify the human SOX2 and U6 genes were as follows: SOX2 forward primer: 5′-GCCGAGTGGAAACTITTGICG-3′ and reverse primer: 5′-GGCAGCGTGTACTIATCCTICI-3′; U6 forward primer: 5′-CTCGCTTCGGCAGCACA-3′ and reverse primer: 5′-AACGCTTCACGAATTTGCGT-3′. Samples were cycled under the following conditions: 40 cycles of 95 °C for 3 min, 95 °C for 20s, 56 °C for 20s, and 72 °C for 30 s. Relative gene expression levels were calculated using the comparative CT method (ΔΔCT) and enrichment was calculated as 2^-[ΔCT (sample) - ΔCT (calibrator)]^.

### Total cellular protein extraction and western blotting

Cultured cells were washed with cold phosphate-buffered saline and treated with radioimmunoprecipitation assay lysis buffer for 10 min at 4 °C. After heating at 100 °C for 10 min, the samples were centrifuged at 14,000 × g for 4 ℃ for 10 min. Approximately 20 µg of each protein sample was separated by 10% sodium dodecyl sulfate-polyacrylamide gel electrophoresis and then transferred to a polyvinylidene fluoride (PVDF) membrane. The membranes were incubated at room temperature with 5% skim milk powder for 1 h, and then with primary antibodies overnight at 4 °C. After washing the PVDF membrane, rabbit or rat secondary antibodies were then applied at a dilution of 1:5,000 in 1× Tris-buffered saline with 0.1% Tween 20 (TBST) for 1 h at room temperature. The membranes were then washed six times with 1× TBST for 5 min and the bands were visualized using an enhanced chemiluminescence reagent. After the membrane surface was evenly covered with the color solution, images were captured and analyzed using chemiluminescence imaging analysis software.

### Cell migration and invasion assay

Transwell assays were performed using transwell inserts (Corning Inc., Corning, NY, USA) containing 8 µm permeable wells, according to the manufacturer's protocol. Transfected PANC1, PaTu8988, and MIA-PaCa-2 cells were harvested, resuspended in serum-free medium, and transferred to 8 µm permeable wells (100,000 cells per well). The cells were then incubated in culture medium containing 10% FBS for 24 h before detection. The cells on the upper surface were scraped off and the migrating cells on the lower surface were fixed and stained with 0.05% crystal violet for 30 min. Five independent fields per transwell were counted, and the average number of cells per field is shown. To assess cell invasion, 100,000 cells were seeded on Matrigel-coated transwell inserts (BD Biosciences, Franklin Lakes, NJ, USA) in serum-free medium. The cells were treated in a manner similar to the cells used in the cell migration assays.

### Colony formation assay

Stable cell lines were collected, resuspended in medium, transferred to six-well plates (500 cells/well), and cultured for 10-14 days until large colonies appeared. The cells were fixed in 4% paraformaldehyde for 15 min and stained with 0.05% crystal violet for 30 min to count the number of colonies formed.

### Cell proliferation assays

Cell proliferation was detected by cell counting kit-8 (CCK-8, Beyotime Institute of Biotechnology, Shanghai, China). For CCK-8 assay, 2 × 104 cells were seeded in 96-well plates for 24 h. At 0, 1, 2, 3, 4, 5, and 6 days after transfection, 10 μl cell counting kit solution was added to each well. 96-well plates were incubated at 37 ° C for 2h, and absorbance values at each time point were measured at 450nm using a microplate reader. All experiments were performed with at least three biological replicates.

### Statistical analysis

All statistical analyses were performed using R software (version 4.2.1). Wilcoxon's rank-sum test and paired-sample Student's t-tests were used to assess the statistical significance of SOX2 expression in unpaired and paired tissues. Associations between clinical features and SOX2 expression levels were assessed using Wilcoxon's rank-sum test and logistic regression analysis. All tests were two-tailed, and p-values < 0.05 were considered statistically significant.

## Results

### The expression of SOX2 in different cancer types

**Figure 1:**
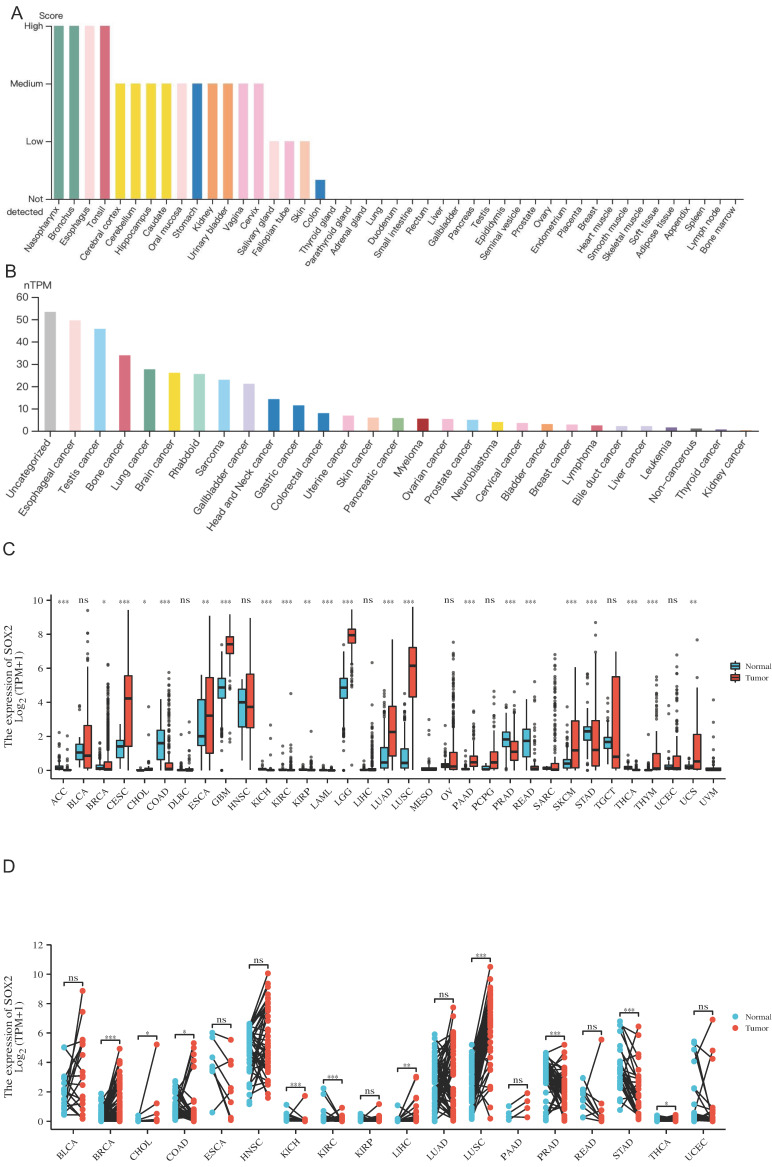
The expression level of SOX2 gene in tumors and normal tissues. A: The expression of SOX2 in normal tissue. B: The expression of SOX2 in cell lines C and D: Pan-cancer analysis of SOX2 from TCGA and GETx database between tumors and adjacent normal tissues.

We explored the expression levels of SOX2 in normal tissues from the GETx database and found that SOX2 was highly expressed in the nasopharynx, bronchus, esophagus, and tonsils (Figure 1A). SOX2 was highly expressed in esophageal cancer (Figure 1B). In the TCGA database, when comparing the expression levels of SOX2 between tumor tissue and adjacent normal tissues, SOX2 was found to be highly expressed in cervical squamous cell carcinoma and endocervical adenocarcinoma (CESC), esophageal carcinoma (ESCA), glioblastoma multiforme (GBM), lower grade glioma (LGG), lung adenocarcinoma (LUAD), lung squamous cell carcinoma (LUSC), PAAD, sarcoma (SARC), skin cutaneous melanoma (SKCM), thymoma (THYM), uterine corpus endometrial carcinoma (UCEC), and uterine carcinosarcoma (UCS), and expressed at low levels in bladder urothelial carcinoma (BLCA), colon adenocarcinoma (COAD), prostate adenocarcinoma (PRAD), and rectum adenocarcinoma (READ; Figure 1C). In addition, SOX2 was highly expressed in breast invasive carcinoma (BRCA), cholangiocarcinoma, colon adenocarcinoma (COAD), kidney chromophobe, liver hepatocellular carcinoma (LIHC), lung squamous cell carcinoma (LUSC), and uterine corpus endometrial carcinoma (UCEC), whereas its expression levels were low in kidney renal clear cell carcinoma (KIRC) and stomach adenocarcinoma (STAD) (Figure 1D).

### Association of SOX2 expression level with molecular or immune cancer subtypes

**Figure 2:**
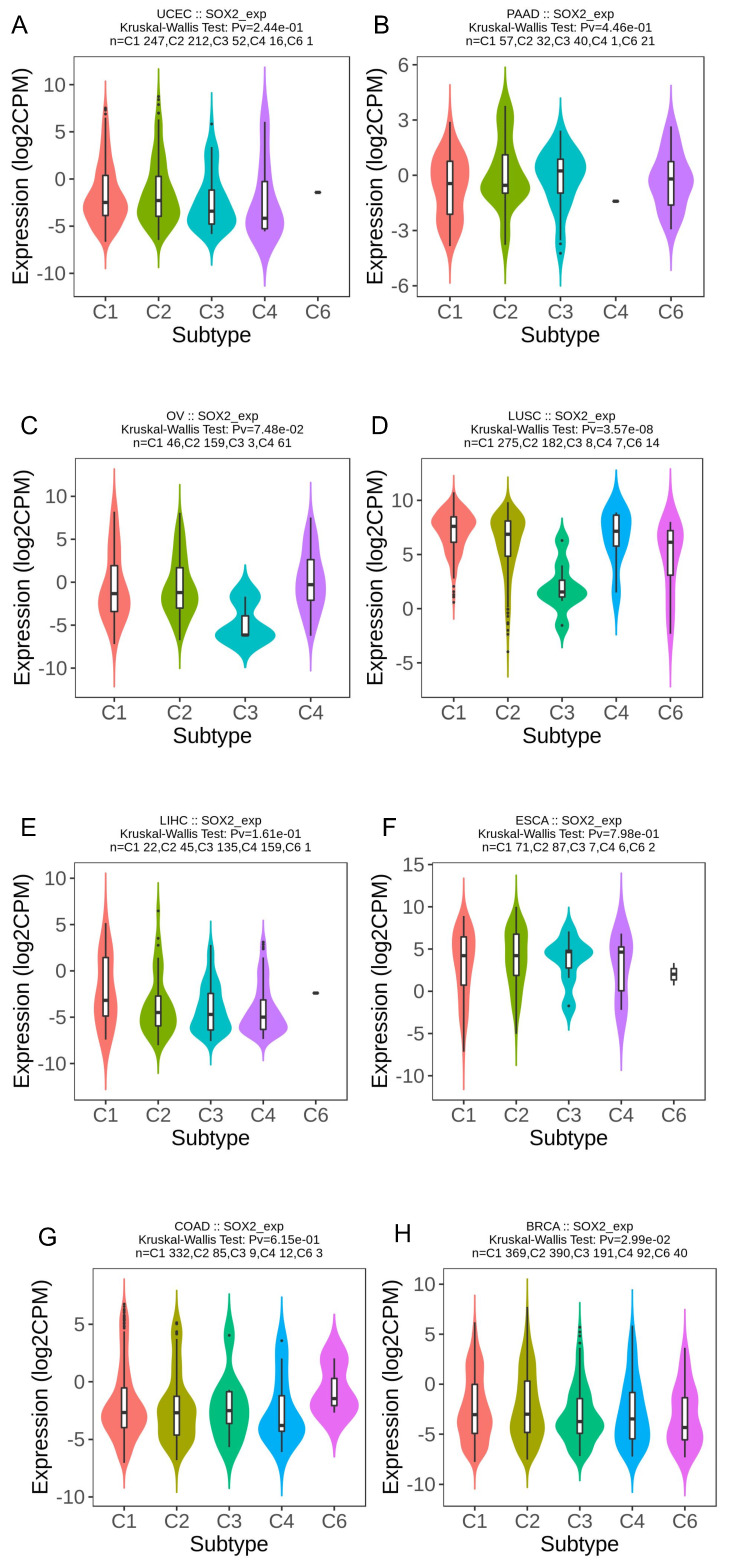
Correlations between SOX2 expression and molecular subtypes across TCGA tumors. A: UCEC. B: PAAD. C: OV. D: LUSC. E: LIHC. F: ESCA. G: COAD. H: BRCA.

**Figure 3:**
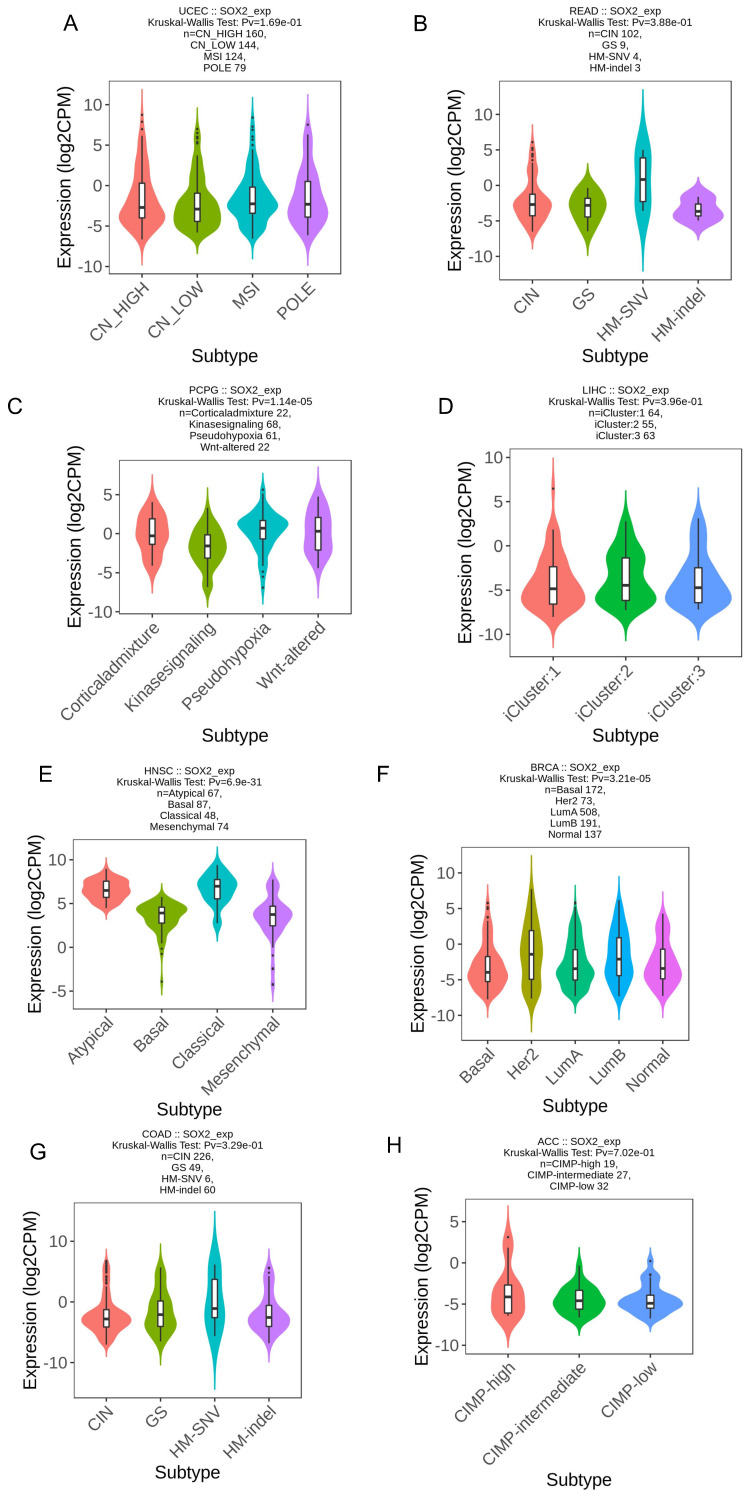
Correlations between SOX2 expression and immune subtypes across TCGA tumors. A: UCEC. B: READ. C: PCPG. D: LIHC. E: HNSC. F: BRCA. G: COAD. H: ACC.

We explored the correlation between SOX2 expression levels and the molecular subtypes of different cancers. SOX2 was differentially expressed in the following tumors: UCEC (C1 had the highest expression level; Figure 2A), PAAD (C3 had the highest expression level; Figure 2B), ovarian serous cystadenocarcinoma (C4 had the highest expression level; Figure 2C), LUSC (C3 had the lowest expression level; Figure 2D), LHC (C1 had the highest expression level; Figure 2E), ESCA (C6 had the lowest expression level; Figure 2F), COAD (C6 had the highest expression level; Figure 2G), BRCA (C6 had the lowest expression level; Figure 2H). The relationship between SOX2 expression level and immune cancer subtypes was also explored. The results showed that SOX2 was differentially expressed in the following tumors: UCEC (CN_LOW had the lowest expression level; Figure 3A), READ (HM-SNW had the highest expression level; Figure 3B), pheochromocytoma and paraganglioma (kinase signaling had the lowest expression level; Figure 3C), LIHC (iCluster had the highest expression level; Figure 3D), head and neck squamous cell carcinoma (HNSC; mesenchymal had the lowest expression level; Figure 3E), BRCA (basal had the lowest expression level; Figure 3F), COAD (HM-SNW had the highest expression level; Figure 3G), and adrenocortical carcinoma (ACC; CIMO-high had the highest expression level; Figure 3H).

### PPI network, functional enrichment, drug susceptibility and pathway analysis of SOX2

**Figure 4:**
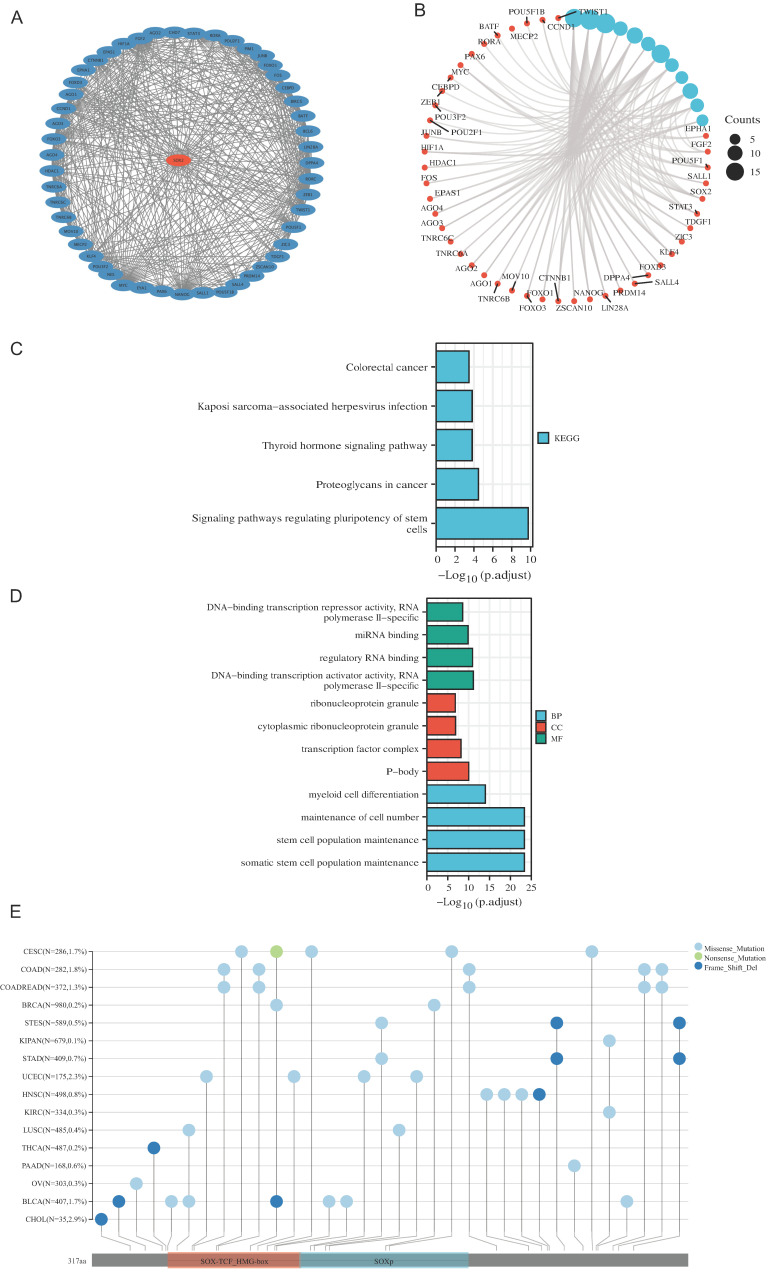
Protein-protein interaction (PPI) network, function enrichment, targeted binding proteins, gene mutation and expression analysis of SOX2 in pan-cancer A. PPI network; B: visual network of GO and KEGG analyses; C: KEGG analysis; D: GO analysis; E. SOX2 gene mutation landscape in pan-cancer.

We screened 50 target binding proteins of SOX2 using the String database and visualized them using Cytoscape (Figure 4A). Furthermore, a looped network diagram was used to display all KEGG and GO enrichment-related molecules and pathways (Figure 4B). Subsequently, we further clarified the results of the KEGG and GO enrichment analyses of SOX2 and its target-binding proteins using bar graphs. KEGG enrichment identified terms mainly related to colorectal cancer, Kaposi-sarcoma-associated herpesvirus infection, thyroid hormone signaling pathway, proteoglycans in cancer, and signaling pathways regulating the pluripotency of stem cells (Figure 4C). The GO enrichment results showed that the primary biological processes were DNA-binding transcriptional repressor activity, RNA polymerase II specificity, miRNA binding, regulatory RNA binding, DNA-binding transcription activator activity, and RNA polymerase II specificity. The cellular components were mainly enriched in ribonucleoprotein granules, cytoplasmic ribonucleoprotein granules, transcription factor complexes, and P-bodies. The molecular functions were primarily myeloid cell differentiation, maintenance of cell numbers, stem cell population maintenance, and somatic stem cell population maintenance (Figure 4D). These results suggest that SOX2 may be involved in the process of cellular and in vivo immunity. The mutation landscape of SOX2 in different cancers was analyzed, and the results showed that the differential expression of SOX2 in SOX-TCF_HMG-box and SOXp was mainly related to missense mutations (Figure 4E). The SOX2-related proteins, EIF24C, TNRC6C, and CCND1, were associated with multiple drugs (Figure S3A). Pathway analysis showed that SOX2 and its related genes play important roles in a variety of biological processes, and may activate or inhibit specific pathways across 32 different cancer types (Figure S3B).

### MIS and MATH analysis of the SOX2 mutation landscape

For pan-cancer MATH analysis of SOX2 expression levels, we calculated the Pearson's correlation coefficient for each tumor, and we observed a significant correlation in 11 tumors (Figure S4A), including a significant positive correlation in the following 10 tumors: GBMLGG (n = 649, r = 0.112110067796125, p = 0.00424213879968673), LUAD (n = 508, r = 0.144776488400433, p = 0.00106671062055533), BRCA (n = 649, r = 0.080543075234121, p = 0.0116597567268291), STES (n = 589, r = 0.214005749642795, p = 1.61328959112151e-7), STAD (n = 409, r = 0.1 95044554612448, p = 0.0000731589038280727), HNSC (n = 498, r = 0.179232526743863, p = 0.000057631766097071), LUSC (n = 485, r = 0.140872362127919, p = 0.00187145243286588), OV (n = 303, r = 0.254853258169487, p = 0.00000704700252692909), testicular germ cell tumor (TGCT; n = 143, r = 0.240905430858214, p = 0.00375146349686758), and ACC (n = 77, r = 0.256926735074619, P = 0.0240921330061556). A negative correlation was observed for PRAD (n = 492, r = -0.134639928728416, p = 0.00276704187307369). For the pan-cancer MSI analysis of SOX2, we calculated the Pearson's correlation coefficient for each tumor type. We observed significant correlations in seven tumor types, including significant positive correlations in the following four tumor types: GBMLGG (n = 657, r = 0.0998288823432262, p = 0.0104569556497638), LUAD (n = 511, r = 0.121327078656369, p = 0.00603137692739204), SARC (n = 10, r = 0.15340604220829, p = 0.01478578506403), and TGCT (n = 148, r = 0.202393158595782, p = 0.0136277618664872). The tumor types with negative correlations were STES (n = 592, r = -0.376087876403097, p = 2.50452918820742e-21), STAD (n = 412, r = -0.412211584037831, p = 2.4826739370725e-18), and PRAD (n = 495, r = -0.412211584037831, p = 2.4826739370725e-18; Figure S4B).

### Prognostic value of SOX2 expression level in different cancer types

**Figure 5:**
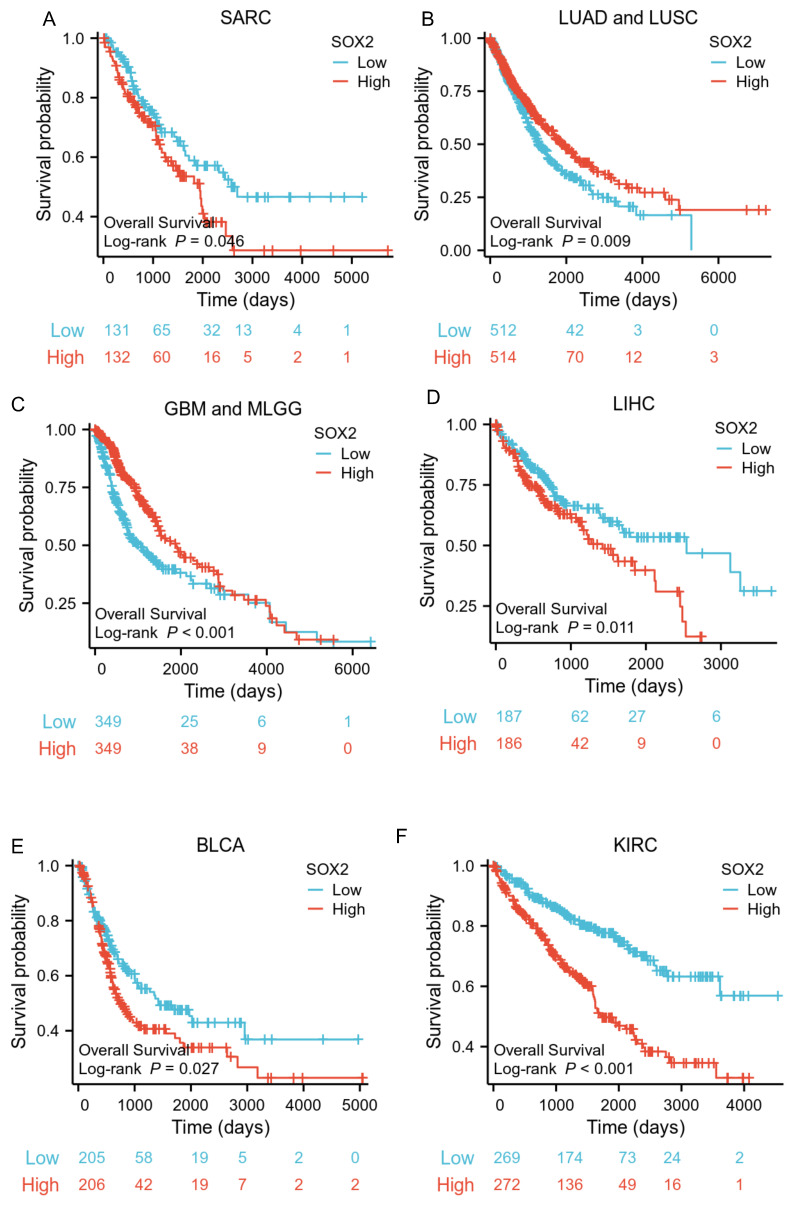
Correlations between SOX2 expression and the prognosis (OS) of cancers. A: SARC. B: LUAD and LUSC. C: GBM and MLGG. D: LIHC. E: BLCA. F: KIRC.

**Figure 6:**
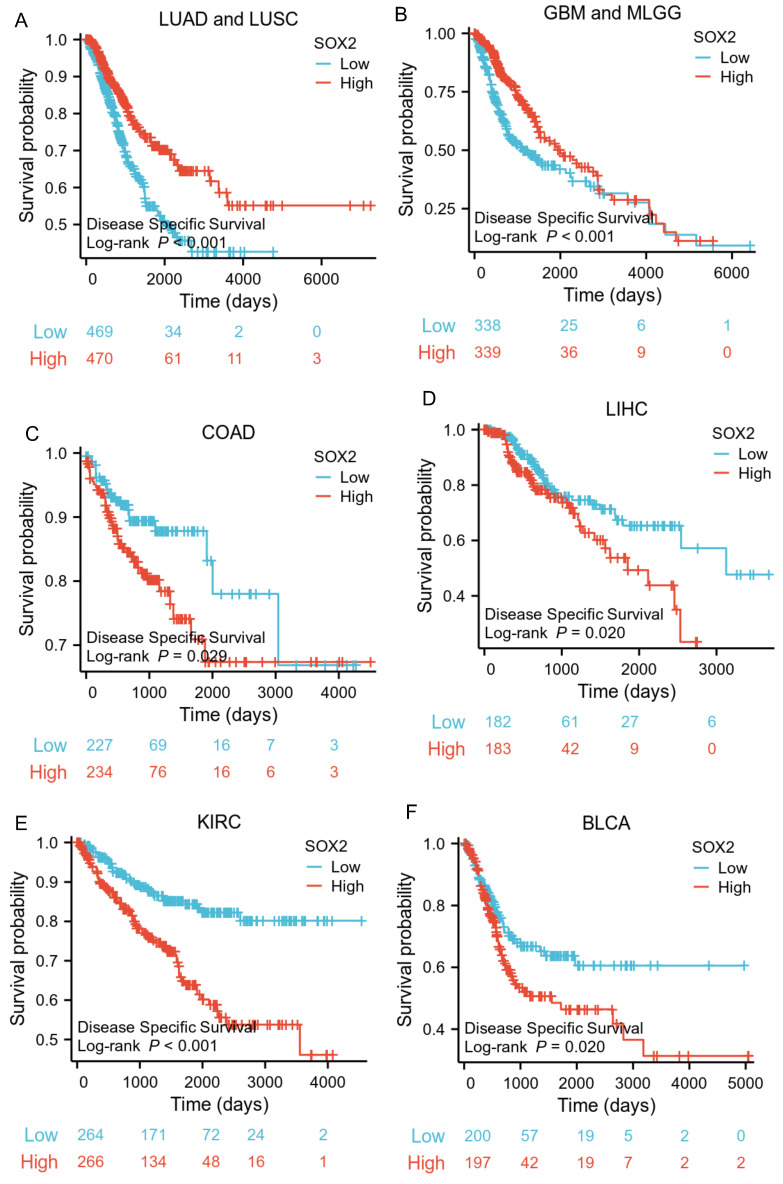
Correlations between SOX2 expression and the prognosis (DSS) of cancers. A: LUAD and LUSC. B: GBM and MLGG. C: COAD. D: LIHC. E: KIRC. F: BLCA.

**Figure 7:**
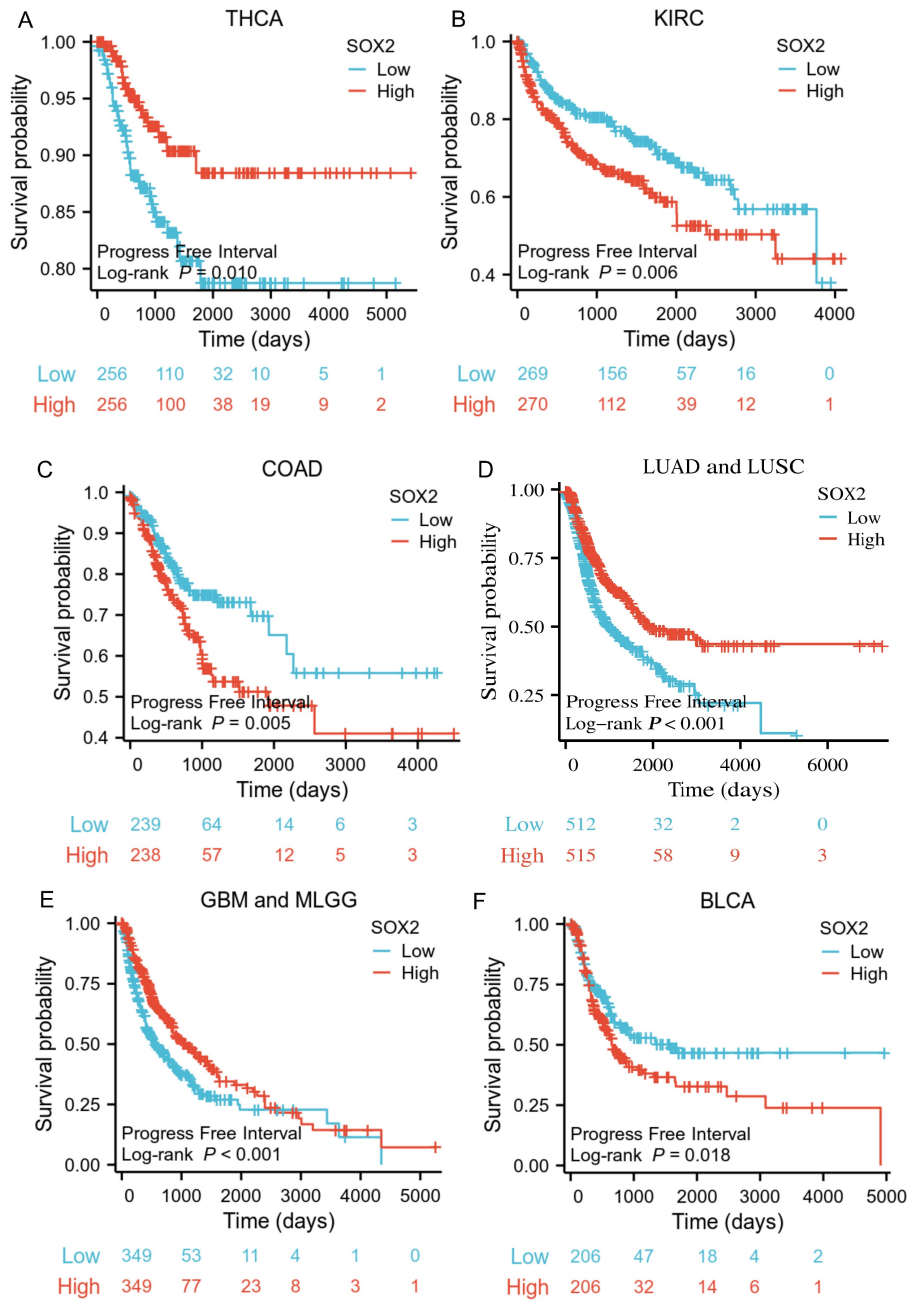
Correlations between SOX2 expression and the prognosis (PFI) of cancers. A: THCA. B: KIRC. C: COAD. D: LUAD and LUSC. E: GBM and MLGG. F: BLCA.

The analysis showed that the SOX2 expression level was closely related to OS, disease-free survival (DFS), and PFI in SARC, LUAD, LUSC, GBM, MLGG, LIHC, BLCA, and KIRC. A log-rank test showed that the SOX2 expression level was associated with OS for SARC (p = 0.046; Figure 5A), LUAD and LUSC (p = 0.009; Figure 5B), GBM and MLGG (p < 0.001; Figure 5C), LIHC (p = 0.011; Figure 5D), BLCA (p = 0.027; Figure 5E), and KIRC (p < 0.001; Figure 5F). A log-rank test showed that the SOX2 expression level was associated with DFS for LUAD and LUSC (p < 0.001; Figure 6A), GBM and MLGG (p < 0.001; Figure 6B), COAD (p =0.029; Figure 6C), LIHC (p = 0.020; Figure 6D), KIRC (p < 0.001; Figure 6E), and BLCA (p = 0.020; Figure 6F). A log-rank test showed that the SOX2 expression level was associated with PFI for thyroid carcinoma (THCA; p = 0.010; Figure 7A), KIRC (p = 0.006; Figure 7B), COAD (p = 0.005; Figure 7C), LUAD and LUSC (p < 0.001; Figure 7D), GBM and MLGG (p < 0.001; Figure 7E), and BLCA (p = 0.018; Figure 7F). These results suggest that a higher SOX2 expression level is associated with a worse prognosis for most tumors. However, in LUAD, LUSC, GBM, and MLGG, SOX2 upregulation may indicate a favorable prognosis, as it was associated with better survival.

### Diagnostic value of SOX2 expression level in different cancer types

**Figure 8:**
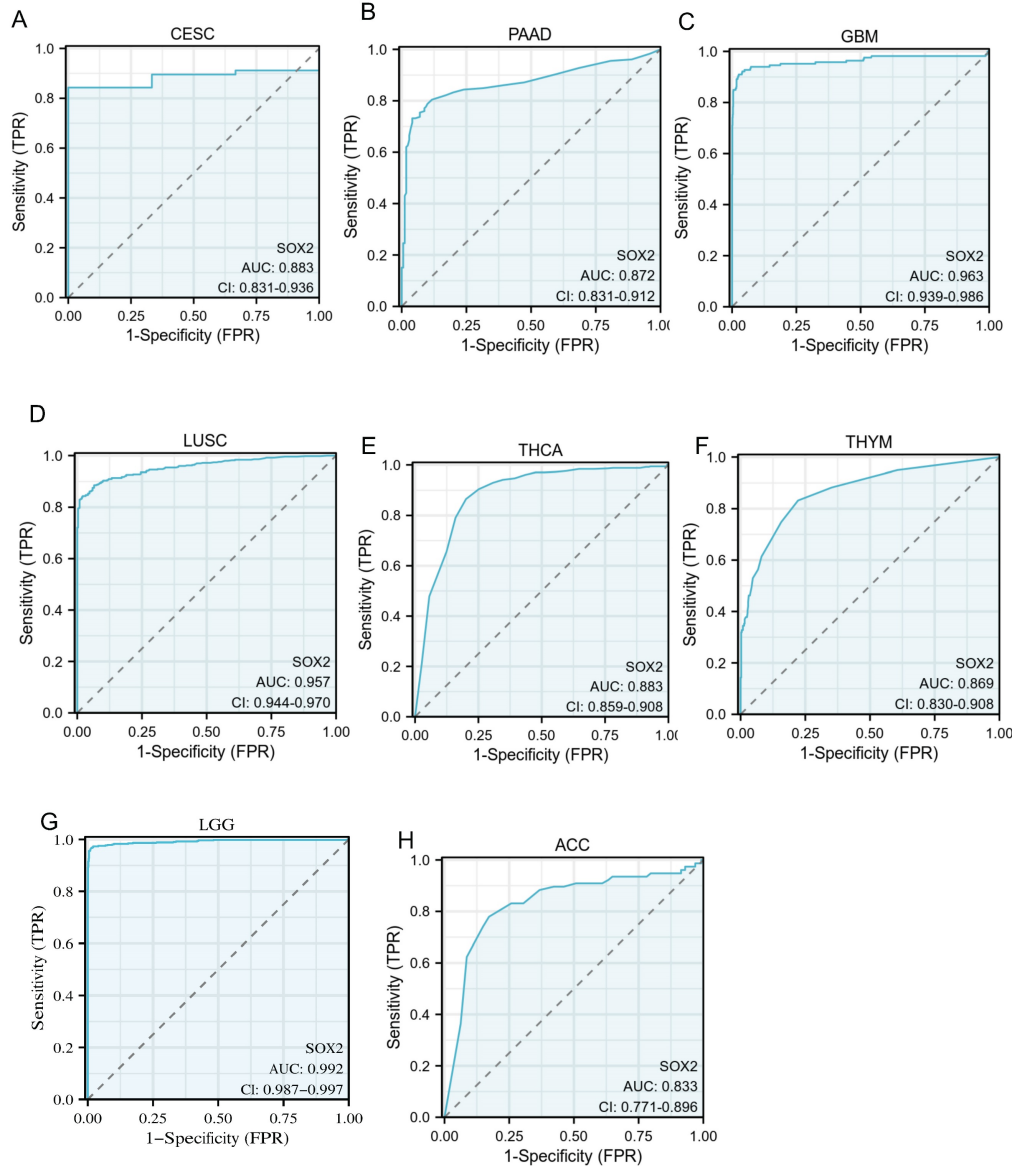
Receiver operating characteristic (ROC) curve for SOX2 expression in pan-cancer. A: CESC. B: PAAD. C: GBM. D: LUSC. E: THCA. F: THYM. G: LGG. H: ACC.

An ROC curve was used to evaluate the diagnostic value of SOX2 for different cancer types, with an AUC > 0.8 indicating excellent predictive performance. We analyzed 33 tumors and found that eight of them had good predictive potential, including CESC (AUC: 0.883, confidence interval [Cl]: 0.831-0.936; Figure 8A), PAAD (AUC: 0.872, Cl: 0.831-0.912; Figure 8B), GBM (AUC: 0.963, Cl: 0.939-0.986; Figure 8C), LUSC (AUC: 0.957, Cl: 0.944-0.970; Figure 8D), THCA (AUC: 0.883, Cl: 0.859-0.908; Figure 8E), THYM (AUC :0.869, Cl: 0.830-0.908; Figure 8F), LGG (AUC: 0.992, Cl: 0.987-0.997; Figure 8G), and ACC (AUC: 0.833, Cl: 0.771-0.896; Figure 8H).

### Clinical significance of SOX2 in pancreatic cancer

**Figure 9:**
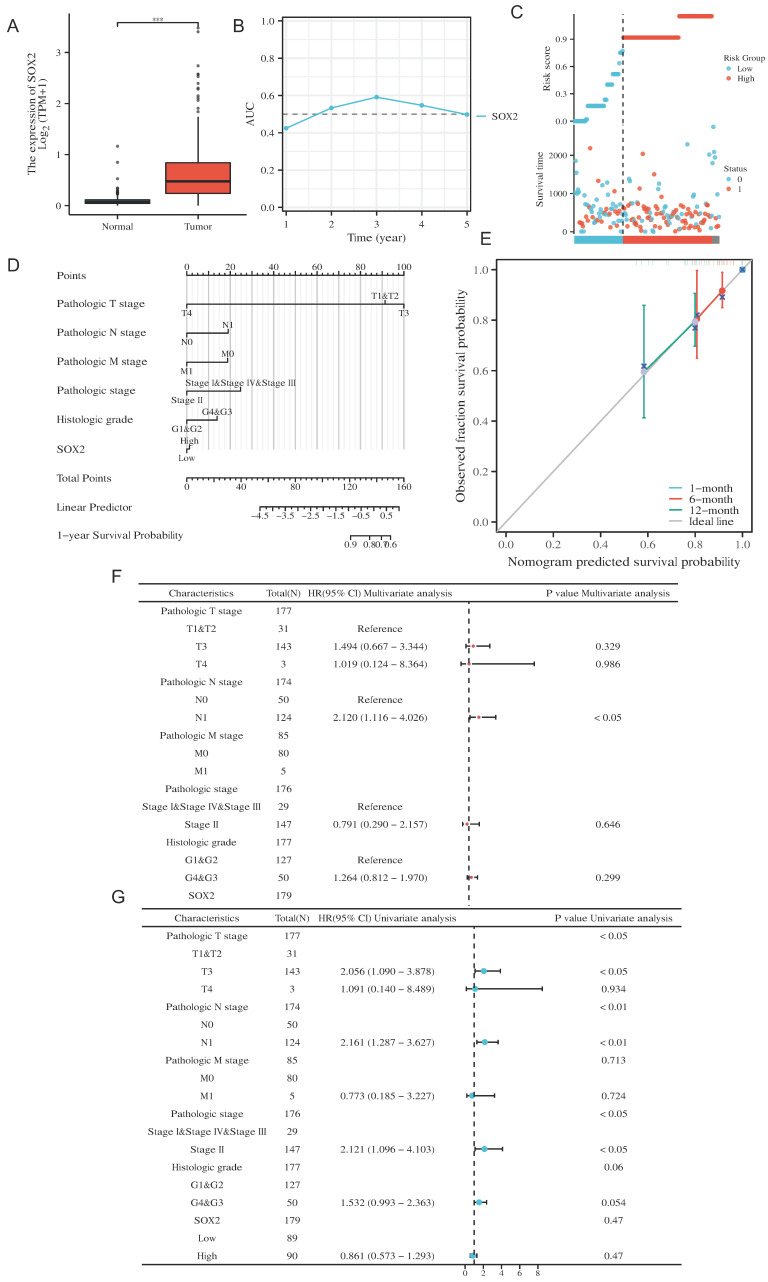
Clinical prognostic analysis of SOX2 expression in pancreatic cancer. A: Relative expression of SOX2 in pancreatic cancer and paracancerous cells. B: The AUC time-dependent curve for SOX2. C: The risk source of SOX2 in PAAD. D: Prognostic Nomogram analysis of SOX2 in PAAD. E: Prognostic Calibration analysis of SOX2 in PAAD. F: The prognostic values of SOX2 expression by univariate analysis. G: The prognostic values of SOX2 expression by multivariate analysis.

We investigated the clinical significance of SOX2 expression in pancreatic cancer. The boxplot showed that, according to the TCGA database, SOX2 was significantly highly expressed in pancreatic cancer (Figure 9A and Figure S1A). The AUC value suggested that the relative expression of SOX2 had predictive power for the 3-year survival rate of patients (Figure 9B). The risk score map suggested that the pancreatic cancer group with a high level of SOX2 expression had a worse prognosis (Figure 9C). Calibration analysis was used to predict the relationship between the SOX2 expression level and prognosis at 1, 6, and 12 months in patients with pancreatic cancer. The prediction results showed a good fit and a high survival rate (Figure 9D). We constructed a nomenclature map based on the independent OS factors to predict the prognosis of pancreatic cancer patients (Figure 9E). Finally, we used univariate and multivariate Cox regression analyses to identify prognostic factors. The results of the univariate analysis demonstrated that SOX2 and N1 (adjusted hazard ratio [HR]: 2.012, 95% CI: 1.116-4.026, p < 0.05) were independent factors predicting OS in patients with PAAD (Figure 9F). The results of the multivariate analysis demonstrated that T3 stage (adjusted HR: 2.056, 95% CI: 1.090-3.878, p < 0.05), N1 stage (adjusted HR: 2.161, 95% CI: 1.287-3.627, p < 0.01), and stage II (adjusted HR: 2.121, 95% CI: 1.096-4.013, p < 0.05) were independent factors predicting OS in patients with PAAD (Figure 9G). These results indicate that SOX2 is a potential biomarker and can be used to predict patient outcomes.

### Analysis of SOX2-related differentially expressed genes and functional enrichment in PAAD

**Figure 10:**
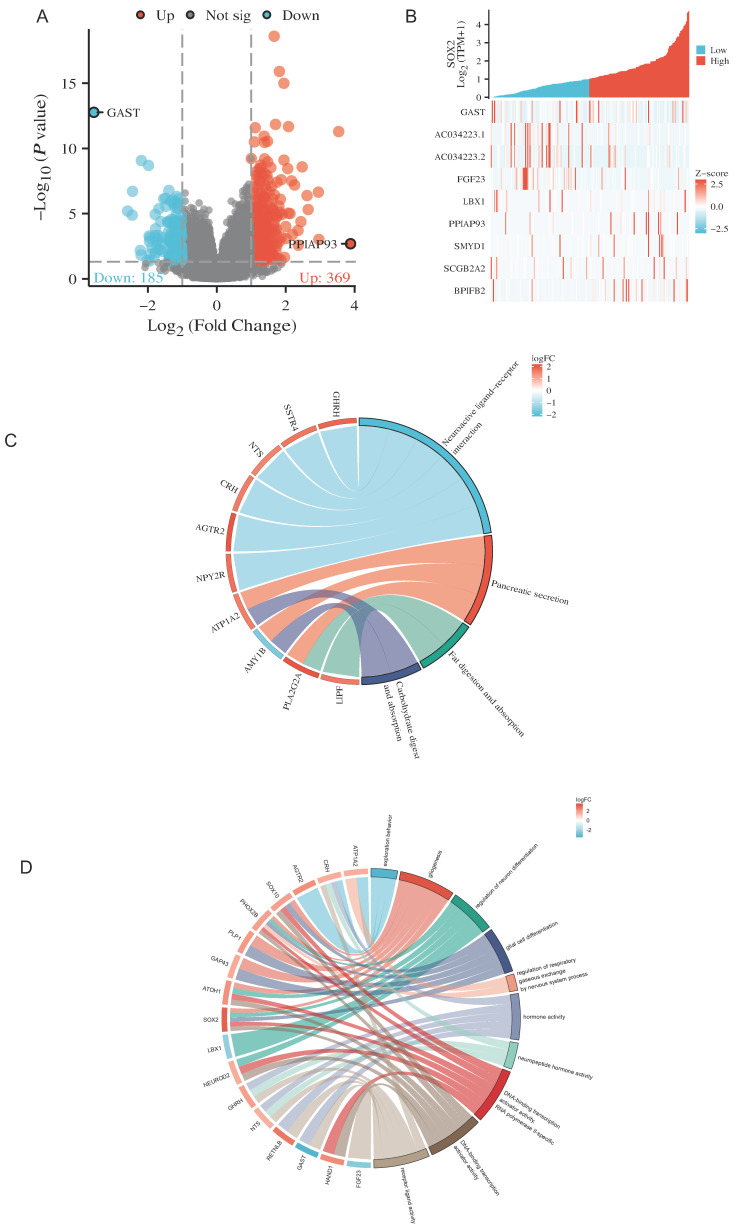
Related differentially expressed genes of SOX2. A and B: The volcano plot which logFc>1.5 and top ten related differentially expressed genes of SOX2 from TCGA database. C and D: KEGG and GO analysis, of SOX2 and their coexpression genes.

The Dseq2 R package was used to analyze the differentially expressed genes (DEGs) related to SOX2 in PAAD. The results showed 106 DEGs between the high-SOX2 -expression and low-SOX2-expression groups, including 74 upregulated genes and 32 downregulated genes (adjusted p < 0.05, |log2-fold change|>1.5; Figure 10A). The relationships between the top five high-expression and the top five low-expression DEGs (including GAST, AC034223.1, AC034223.2, FGF23, LBX1, PPIAP93, SYMD1, SCGB2A2, and BPIFB2) and SOX2 are presented in Figure 10B. In the KEGG enrichment analysis, the DEGs related to SOX2 were mainly enriched in neuroactive ligand-receptor interactions, pancreatic secretion, fat digestion and absorption, and carbohydrate digestion and absorption (Figure 10C). In the GO enrichment analysis, DEGs related to SOX2 were mainly enriched in exploration behavior, gliogenesis, regulation of neuron differentiation, glial cell differentiation, regulation of respiratory gaseous exchange by a nervous system process, hormone activity, neuropeptide hormone activity, DNA-binding transcriptional activator activity, RNA polymerase IL-specific, DNA-binding transcription activator activity, and receptor-ligand activity (Figure 10D). And in KEGG function enrichment, exploration behavior, neuropeptide hormone activity, DNA-binding transcriptional activator activity suggested that it was related to tumor immune infiltration.

### Correlation between methylation and SOX2 expression levels

**Figure 11:**
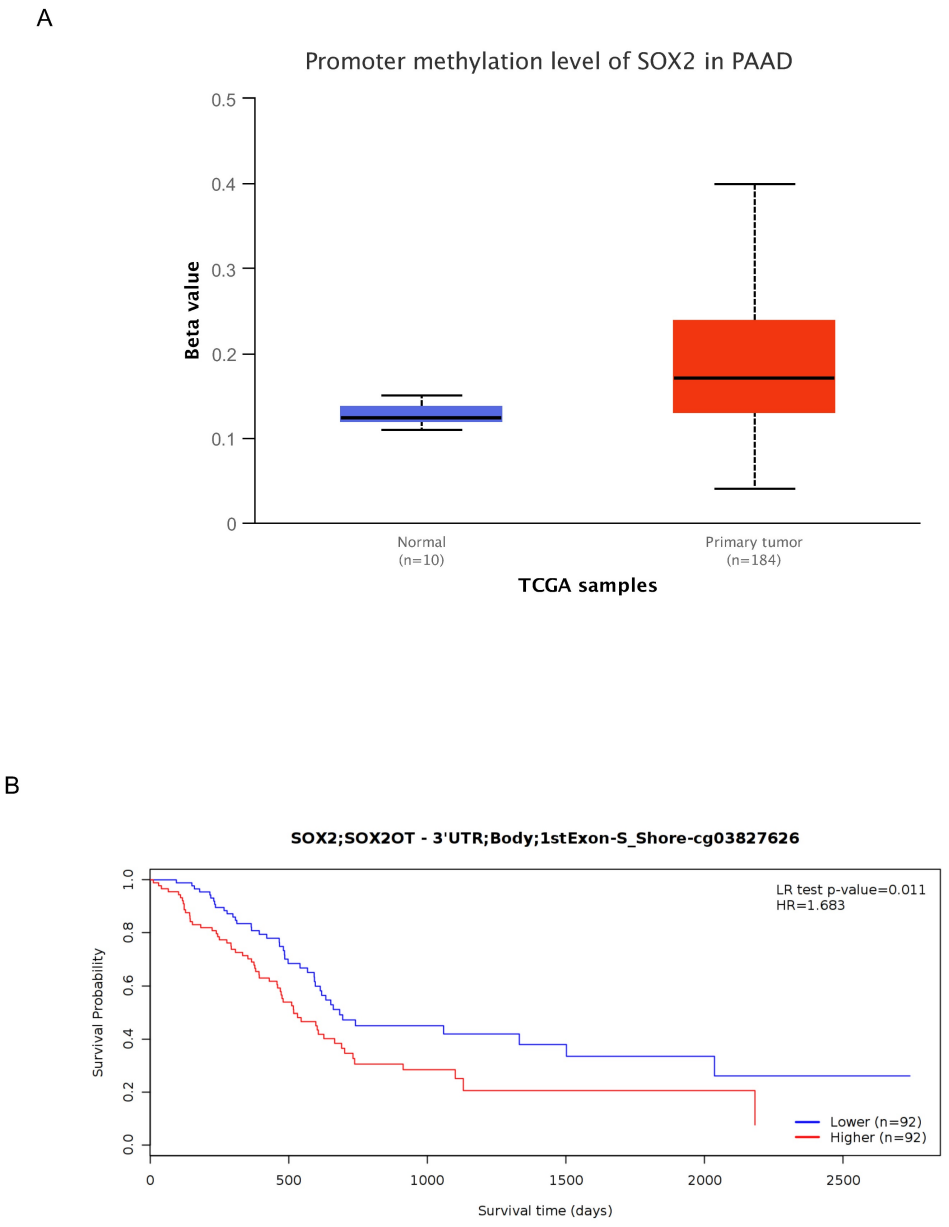
DNA methylation level of SOX2 and its effect on prognosis of patients with PAAD. A: The promoter methylation level of SOX2 in PAAD was obtained from the UALCAN database. B: Kaplan-Meier survival curves for several methylation sites of SOX2.

To further characterize the mechanism underlying SOX2 overexpression in PAAD, we explored the correlation between SOX2 expression levels and methylation status using online tools. We found that most methylation sites in the MCTS1 gene sequence were hypomethylated in breast cancer and that the degree of methylation correlated with the patient outcome. Patients with low levels of SOX2 methylation had longer overall survival than those with high SOX2 methylation levels (Figure S5A). DNA methylation of the SOX2 promoter was significantly lower in PAAD tissue than in normal tissue from the UALCAN database (Figure 11A). In addition, several methylated sites in SOX2, including cg03827625, were associated with a poor prognosis (Figure 11B).

### Correlation between SOX2 expression level and immune infiltration

**Figure 12:**
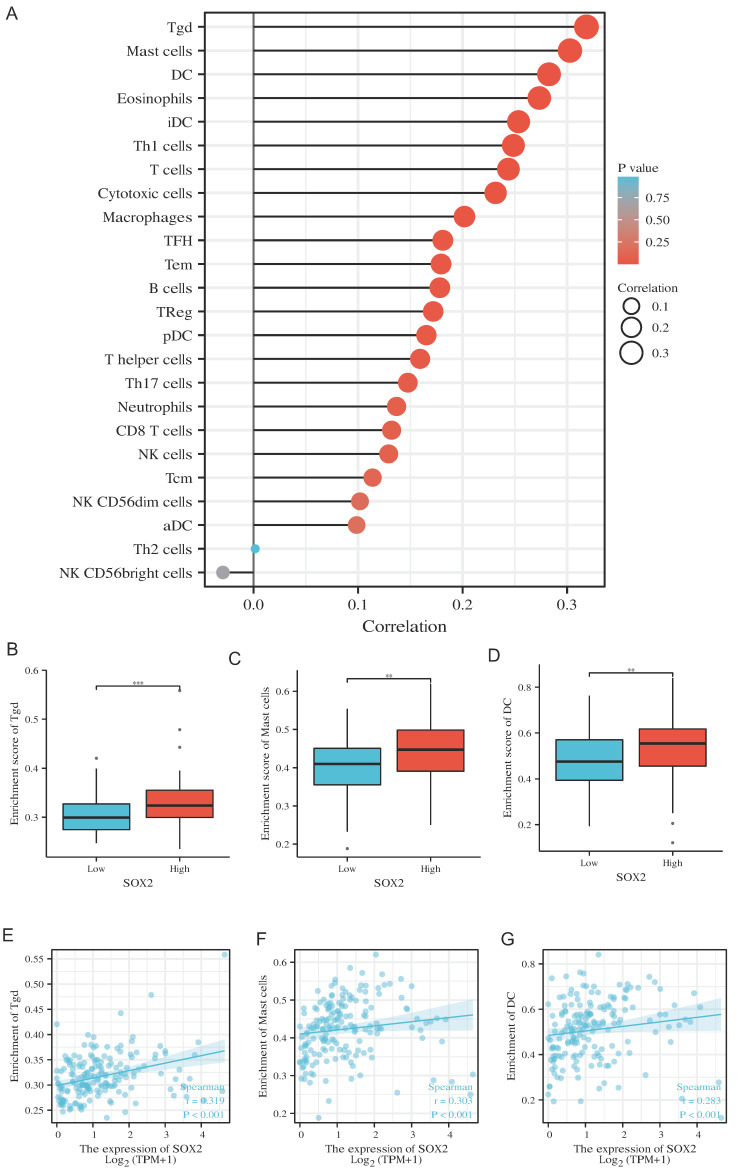
Correlation of SOX2 expression with immune infiltration level in pan-cancer and PAAD. A: Correlation between SOX2 expression and relative abundance of 24 types of immune cell. The size of dot corresponds to the absolute Spearman's correlation coefficient values. B, C and D: Comparison of top3 immune infiltration levels of immune cells between the high- and low-SOX2 expression groups. E, F and G: Correlations between the relative enrichment scores of immune cells (including Tgd, Mast cells and DC) and the expression of SOX2.

Our findings suggest that SOX2 is positively correlated with most immunomodulatory genes and the immune checkpoints in the pan-cancer analysis. In pancreatic cancer, this trend was positively associated with most tumor similarities (Figure S6A and S7A). In pancreatic cancer, high SOX2 expression levels were associated with Tgd, mast cell, and DC infiltration (Figure 12A). The SOX2 expression level was significantly correlated with the number of Tgd cells (r = 0.319, p < 0.001), mast cells (r = 0.303, p < 0.001), and DCs (r = 0.283, p < 0.001; Figure 12B-D). Moreover, the enrichment scores of Tgd cells, mast cells, and DCs were higher in the high-SOX2-expression group than the low-SOX2-expression group (all p < 0.001; Figure 12E-G). At the single-cell level, SOX2 enriched DC and Mast cells mainly in malignant cells in pancreatic cancer. The effect of SOX2 on immune infiltration of pancreatic cancer was further revealed (Figure S2A and 2B).

### Relative SOX2 expression level and migration and invasion ability in pancreatic cancer cell lines

**Figure 13:**
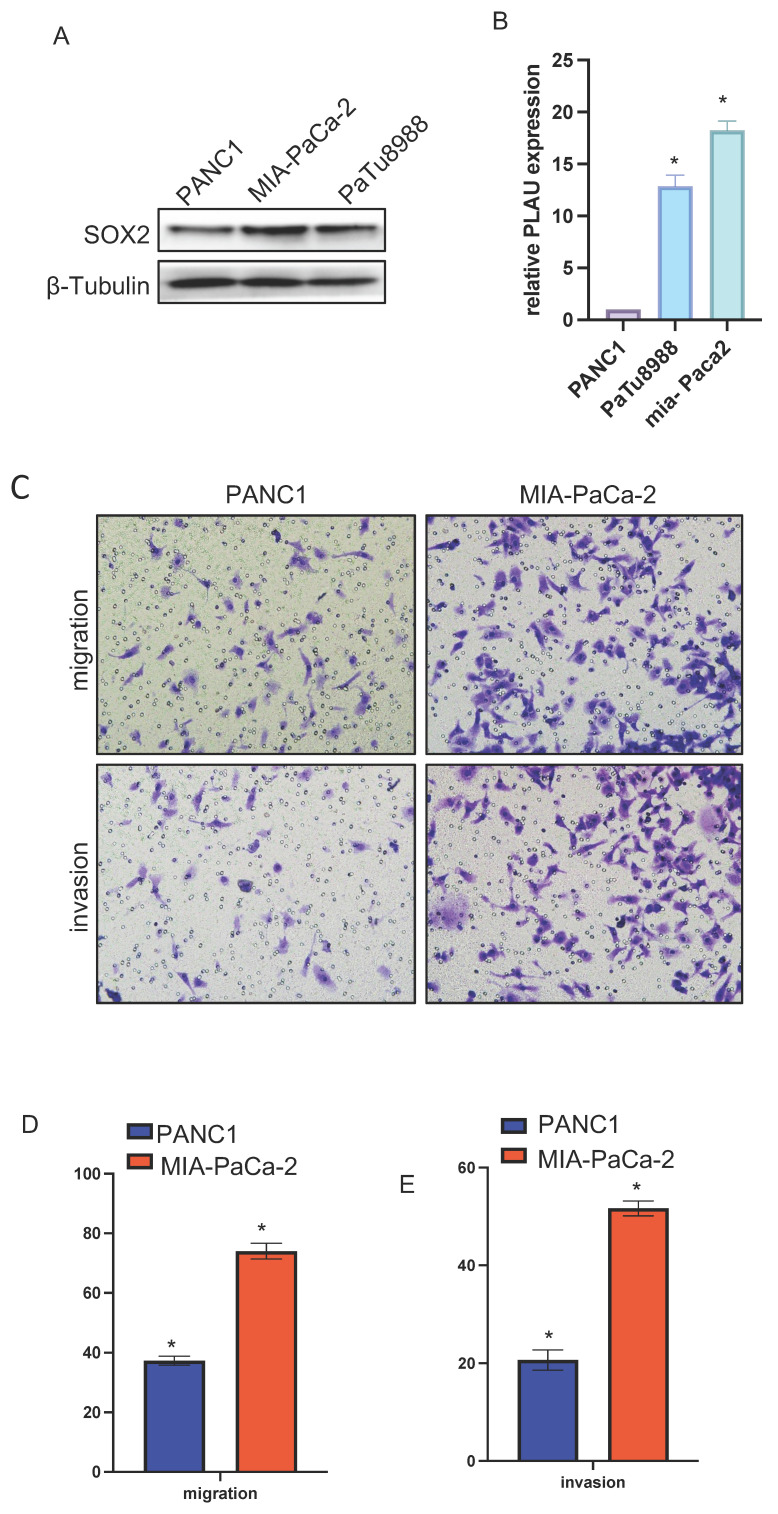
SOX2 relative expression and migration and invasion ability in pancreatic cancer cell lines A and B. The relative expression of SOX2 in pancreatic cancer cell lines C, D and E: Cell migration and invasion assay of SOX2 in Mia-PaCa2 and PANC-1.

**Figure 14:**
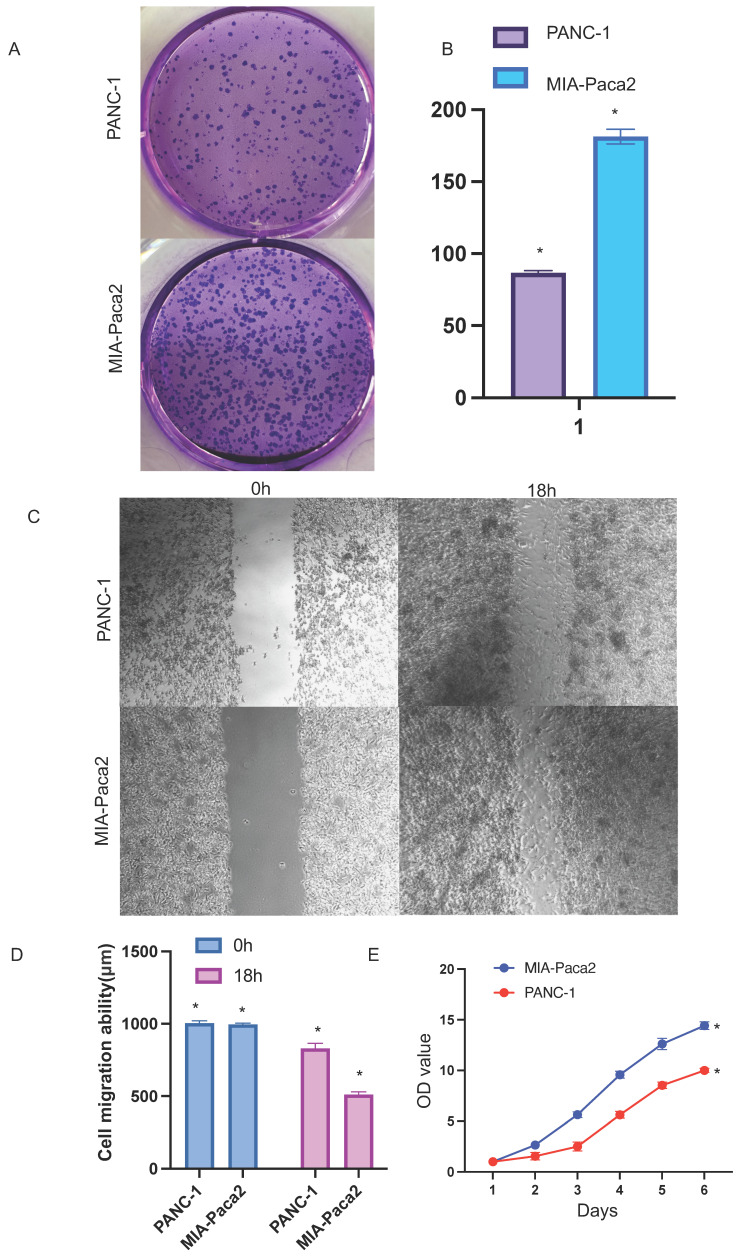
Functional effects of SOX2 in PAAD cells A and B Colony formation assay was performed to compare the effect of NCAPG2 on proliferation. Histograms shows the number of colony formation. C and D: Cell scratch assay detects migration ability and histogram shows the relative area of wound healing. E: Proliferative capacity of SOX2 in PAAD.

We verified the relative expression level of SOX2 in three pancreatic cancer cell lines, PANC1, MIA-PaCa-2, and PaTu-8988, by quantitative PCR and western blotting. The results showed that SOX2 expression was the highest in MIA-PaCa-2 cells and the lowest in PANC1 cells, with PaTu-8988 had intermediate SOX2 expression levels (Figure 13A, B). Since SOX2 expression was highest in MIA-PaCa2 and lowest in PANC-1, we chose to conduct further experiments in MIA-PaCa2 and PANC-1. Transwell assays showed that the migration and invasion abilities of Mia-PaCa2 cells were stronger than those of PANC-1 cells, suggesting that SOX2 plays a significant role in the migration and invasion of pancreatic cancer (Figure 13C-E). Scratch experiments showed that MIA-Paca2 cell lines had a stronger migration ability than PANC1 cells (Figure 14C, D).

### Effect of SOX2 on pancreatic cancer proliferation

CCK-8 experiment was used to further verify the effect of SOX2 on cell proliferation. The cell proliferation ability of PANC-1 group was significantly weaker than that of MIA-Paca2 group (Figure 14E).

### Effect of SOX2 on colon cancer proliferation

Colony-formation assays showed that the proliferation and colony formation ability of MIA-Paca2 cells were stronger than those of PANC-1 cells (Figure 14A, B).

## Discussion

SOX2 is a member of the SOX family and it plays an essential role in pancreatic cancer. Evidence shows that the SOX family plays a crucial role in many cancers 14,17,26,27. For example, SOX4 is considered a significant regulator of EMT in many cancers 13. SOX15 transcription enhances the function of AOC1 to modulate ferroptosis and the progression of prostate cancer 15. Moreover, SOX9, which is activated by FARSA-AS1, promotes the growth, stemness, and metastasis of colorectal cancer 16. For SOX2, in colorectal cancer, it acts together with TM4SF1 to promote tumor EMT and maintain its stemness 33. In lung cancer, CDK1 drives SOX2 to maintain tumor cell stemness 34, and similar conclusions have been made in glioma and breast cancer 35,36.

However, previous studies have mainly focused on tumor stem cells and EMT 18. Some studies have shown that SOX family has potential in immune infiltration in gliomas 19 and hepatocellular carcinoma 12. For SOX2, in non-small cell lung cancer, SOX2 works together with the transcription factor NKX2-1 to reshape the tumor's immune microenvironment 28, while in esophageal cancer and adolescent gliomas, SOX2 is considered a potential immunotherapy target 29, 30. In oral cancer cells, high expression of SOX2 is significantly correlated with PD-L1 and is associated with immune escape 31. These studies indicate that SOX2, in addition to serving as a tumor stem cell indicator 1, is a potential biomarker in immune infiltration and tumor microenvironment shaping. In pancreatic cancer, there is no study related to SOX2 immune infiltration. Therefore, we predicted that SOX2 may have a role in tumor immunology and may be a new therapeutic target in PAAD.

Thus, we decided to analyze SOX2 using pan-cancer analysis, focusing on its significance in pancreatic cancer. In this study, we performed a pan-cancer analysis of SOX2 using the TCGA, GETX, and TSIDB online databases and analyzed the expression of SOX2 in different tissues, tumor subtypes, and immune cells. The results showed that SOX2 was highly expressed in various tumors. STRING and PPI networks were used to explore the related proteins. Functional enrichment analysis showed that they could affect tumors in many ways. Kaplan-Meier plots for OS, DSS, and PFI, and ROC curves showed that SOX2 could act as a novel biomarker and predictor of prognosis in many cancer patients.

These results further validate the clinical potential of SOX2, which is mainly related to OS, tumor stage, and other relevant clinical indicators. In addition, genes co-expressed with SOX2 in pancreatic cancer were explored, and KEGG and GO enrichment indicated that SOX2 was involved in multiple pathways in pancreatic cancer. Methylation analysis showed that SOX2 methylation, including at cg03827625, was significantly different between pancreatic cancer and normal tissue and was associated with a poor prognosis. Finally, we performed a bioinformatics study of SOX2-related immune infiltration in pancreatic cancer and showed that high SOX2 expression levels were positively correlated with Tgd, mast cell, and DC infiltration. δT cells have been shown to suppress αβT cell activation to promote the development of PAAD 20. Mast cells and DCs also play essential roles in many cancers, such as Scf-mediated inflammation enhancement and immunosuppression in the tumor microenvironment 21. However, the underlying mechanisms remain unclear. SOX2 may play a crucial role in these axes, but this needs to be explored further.

To sum up, since immune cells are an important part of the tumor microenvironment (EMT) 32, we believe that SOX2 affects immune invasion by recruiting Tgd, mast cells and DC cells in pancreatic cancer, changes the tumor microenvironment, and ultimately affects the occurrence and development of pancreatic cancer.

This study had several limitations. First, we only explored SOX2 using online databases, such as TCGA and GETX; therefore, more clinical samples are needed. Second, biological experiments are required to validate the findings and provide high-quality evidence. Therefore, further studies will be needed using specific tissues and cells, such as the flow classification of mice after tumor formation, to further validate the bioinformatics predictions. In addition, further validation of the immune infiltration findings using single-cell sequencing is needed after the corresponding patient samples have been obtained and statistically grouped.

In summary, we identified the potential role of SOX2 in pancreatic cancer using pan-cancer analysis and evaluated it using a combination of functional enrichment, methylation, and immune infiltration. These results suggest that SOX2 plays a significant role in many aspects of pancreatic cancer and has potential biological value.

## Supplementary Material

Supplementary figures.
