# Supplementary material for: Pan-Cancer Analysis Reveals SOX2 as a Promising Prognostic and Immunotherapeutic Biomarker Across Various Cancer Types, Including Pancreatic Cancer
Source: J Cancer. 2024 Jan 1;15(1):251–74. doi: 10.7150/jca.88397 (PMC10751676; doi:10.7150/jca.88397)
Supplement: Supplementary file 1 — Supplementary figures. [file jcav15p0251s1.pdf]

A

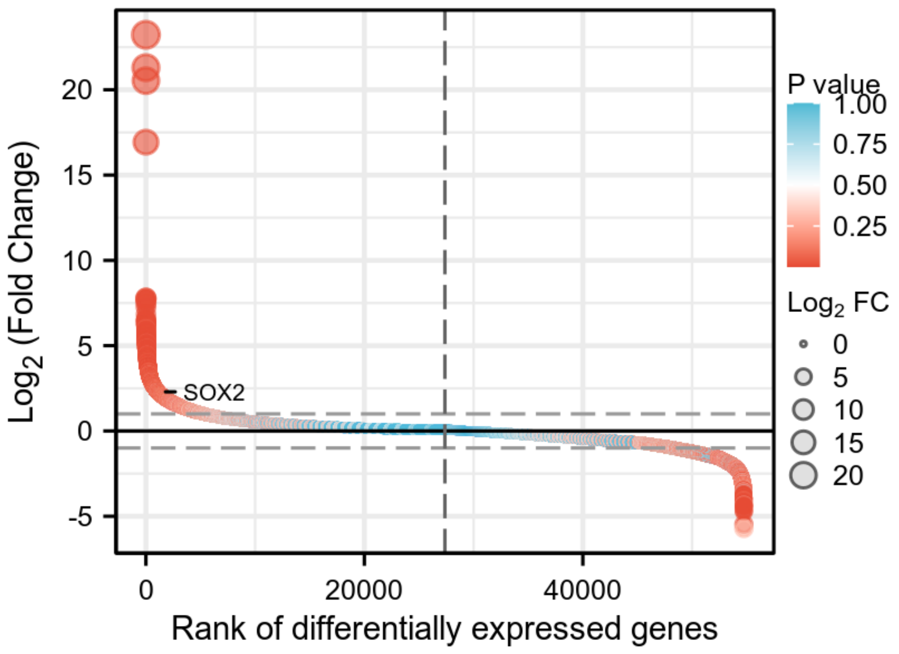

FigureS1: Relative expression of sox2 (A). Relative expression of sox2 in pancreatic cancer

A

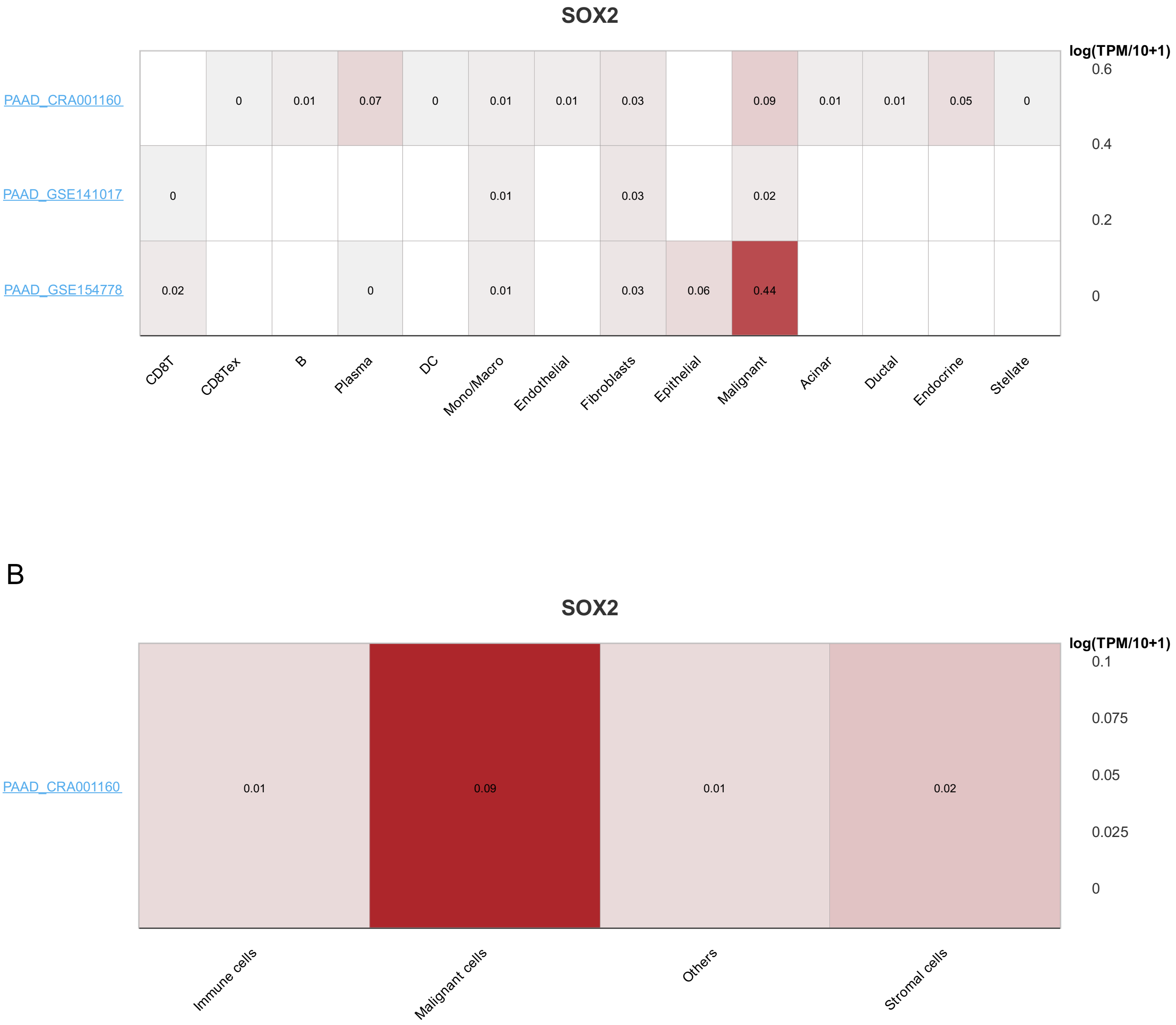

B

FigureS2: Analysis of immune infiltration of DC and Mast cells by SOX2 through single-cell sequencing in pancreatic (A).Mast cell (B).DC cell

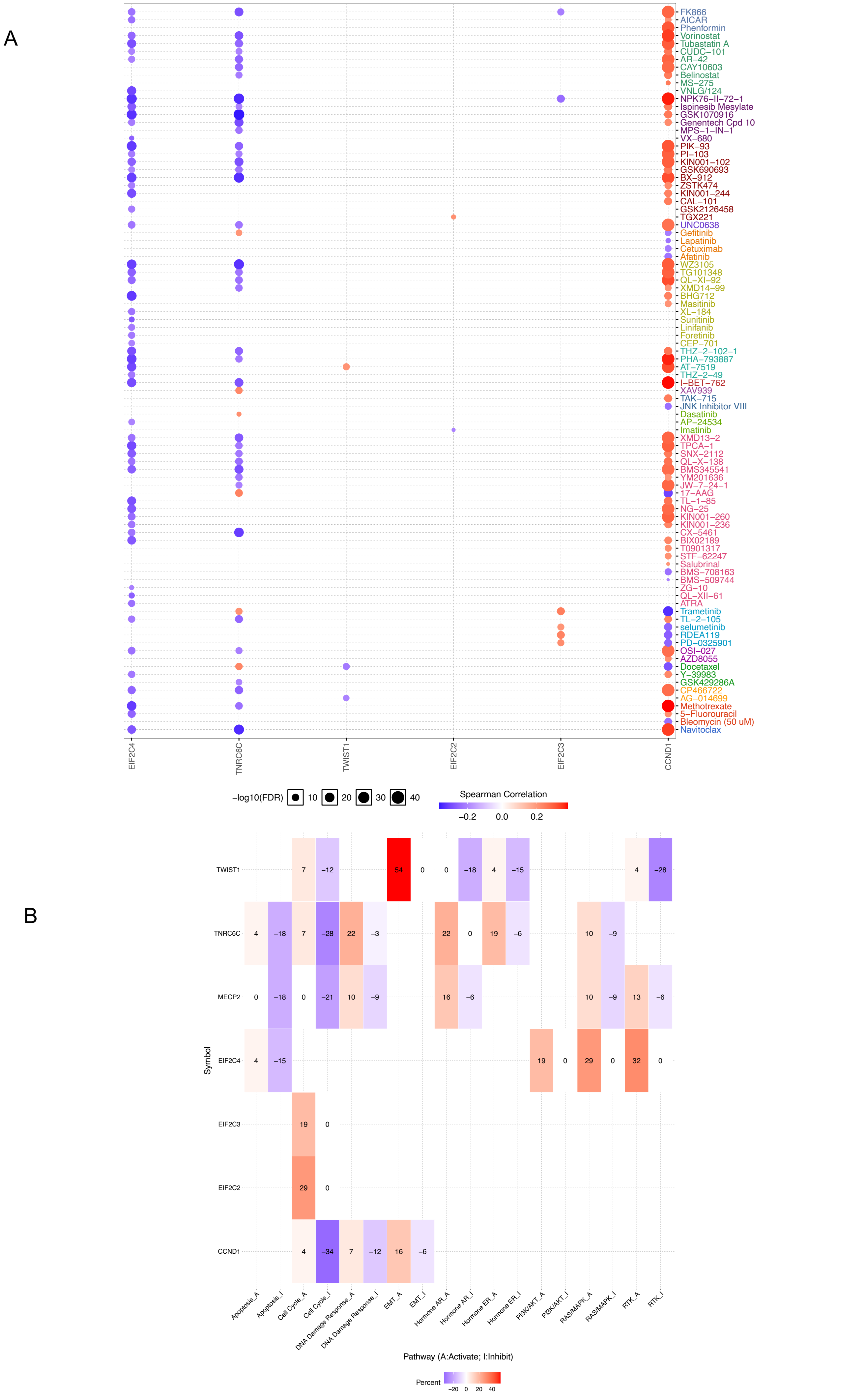

FigureS3:Drug sensitivity and pathway analysis of sox2 in pan-cancer （A）Drug sensitivity analysis of SOX2 in pan-cancer （B）Pathway analysis of sox2 in pan-cancer

A

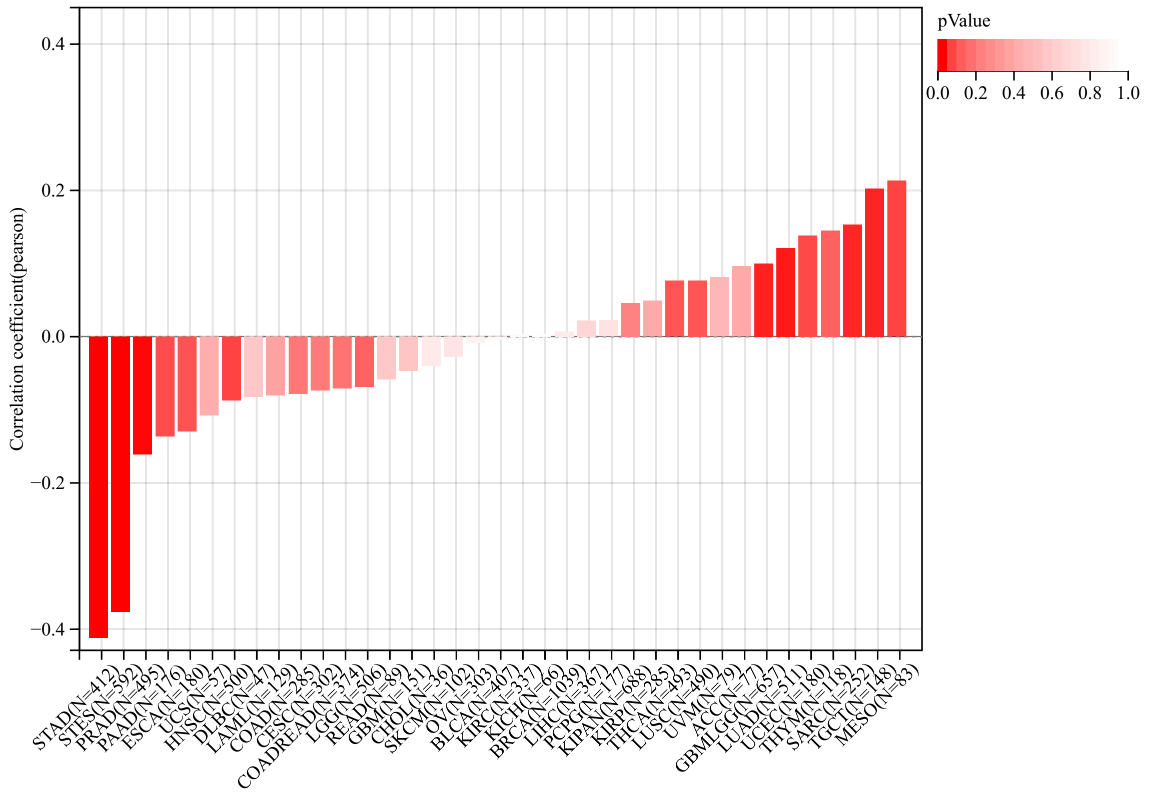

B

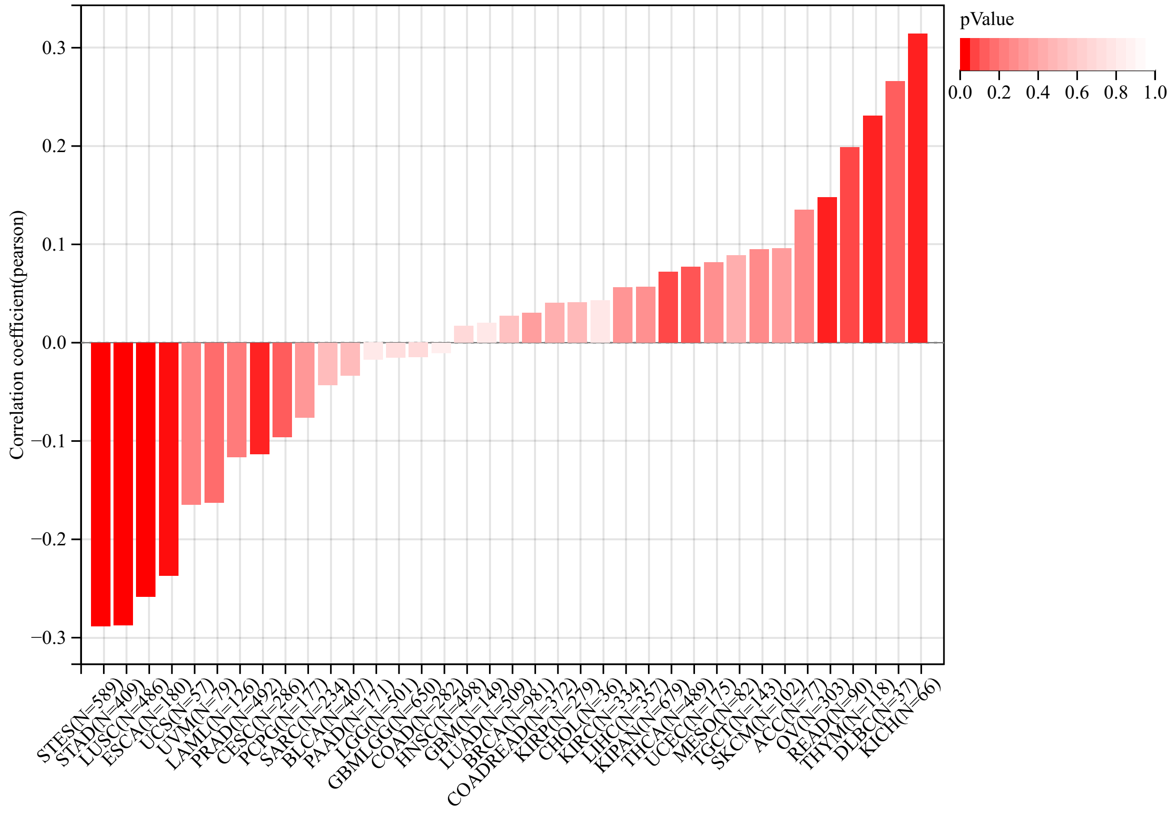

FigureS4:Microsatellite Instability(MSI) and tumor mutational burden(MATH) of SOX2 in pan-cancer analysis.(A)MSI (B)MATH

A

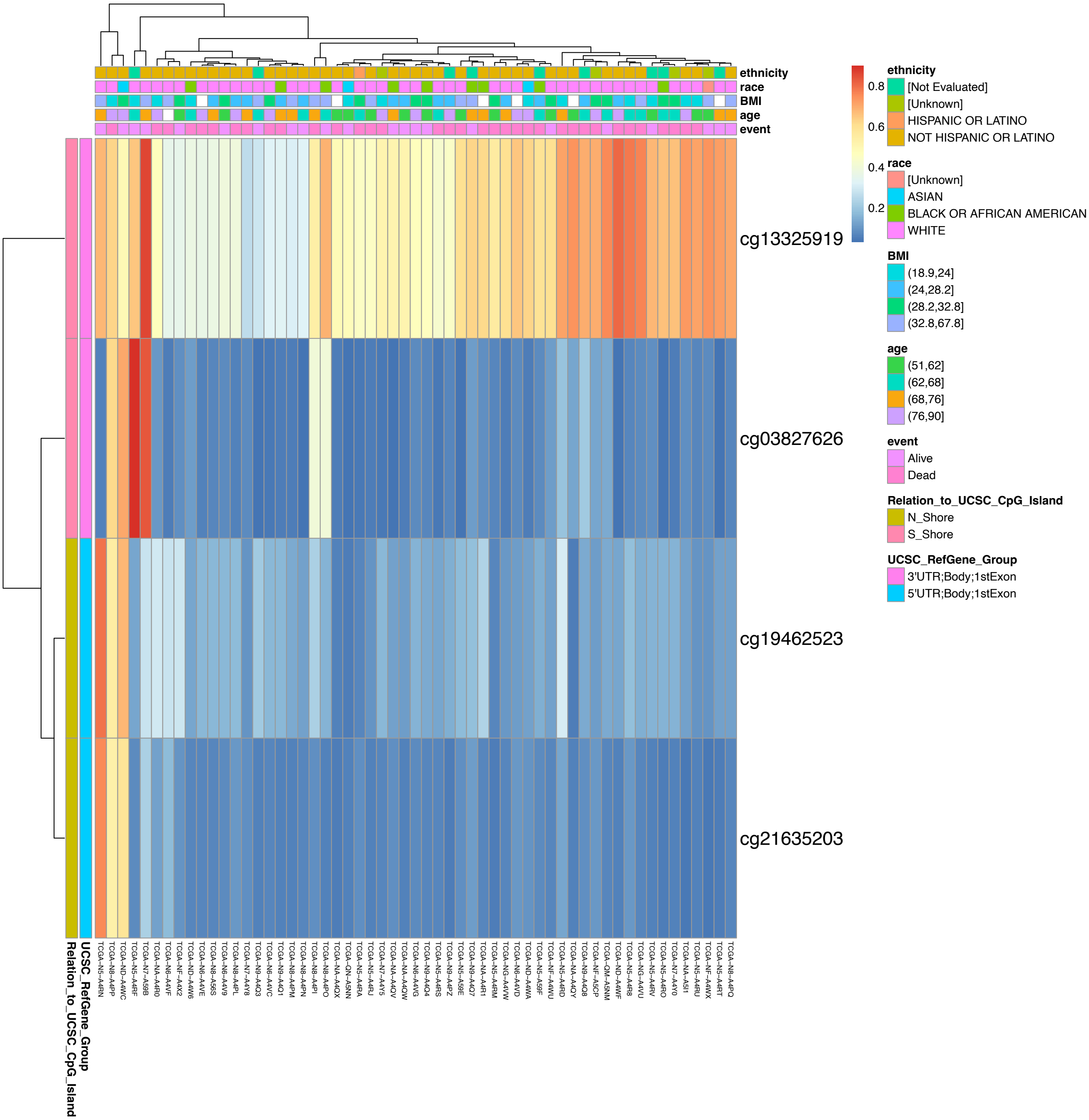

FigureS5:DNA methylation level of SOX2 and its effect on prognosis of patients with PAAD. (A) Correlation between SOX2 mRNA expression level and methylation level.

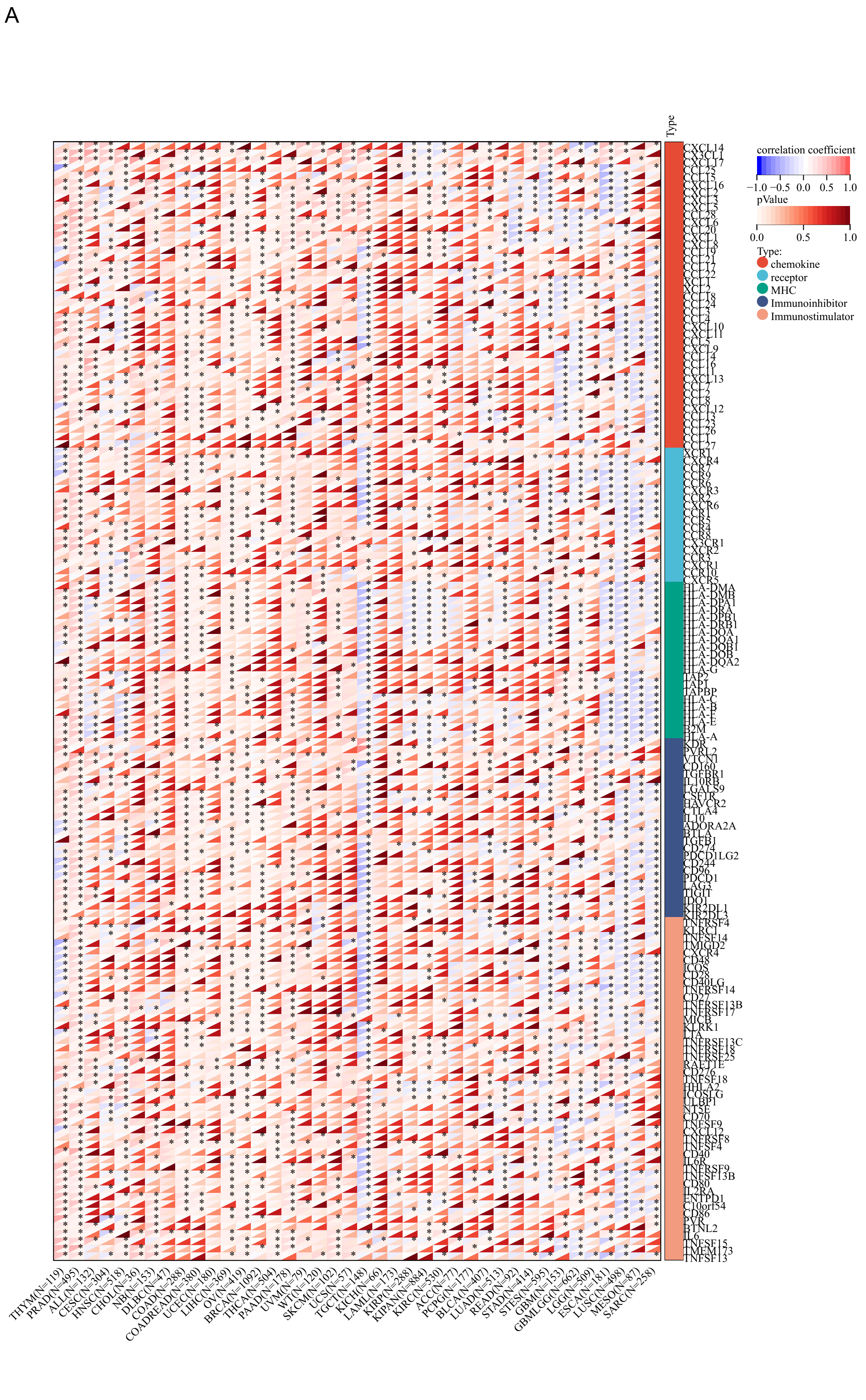

FigureS6; Immunomodulatory gene analysis (A).Immunomodulatory gene analysis of SOX2 in pan-cnacer

A

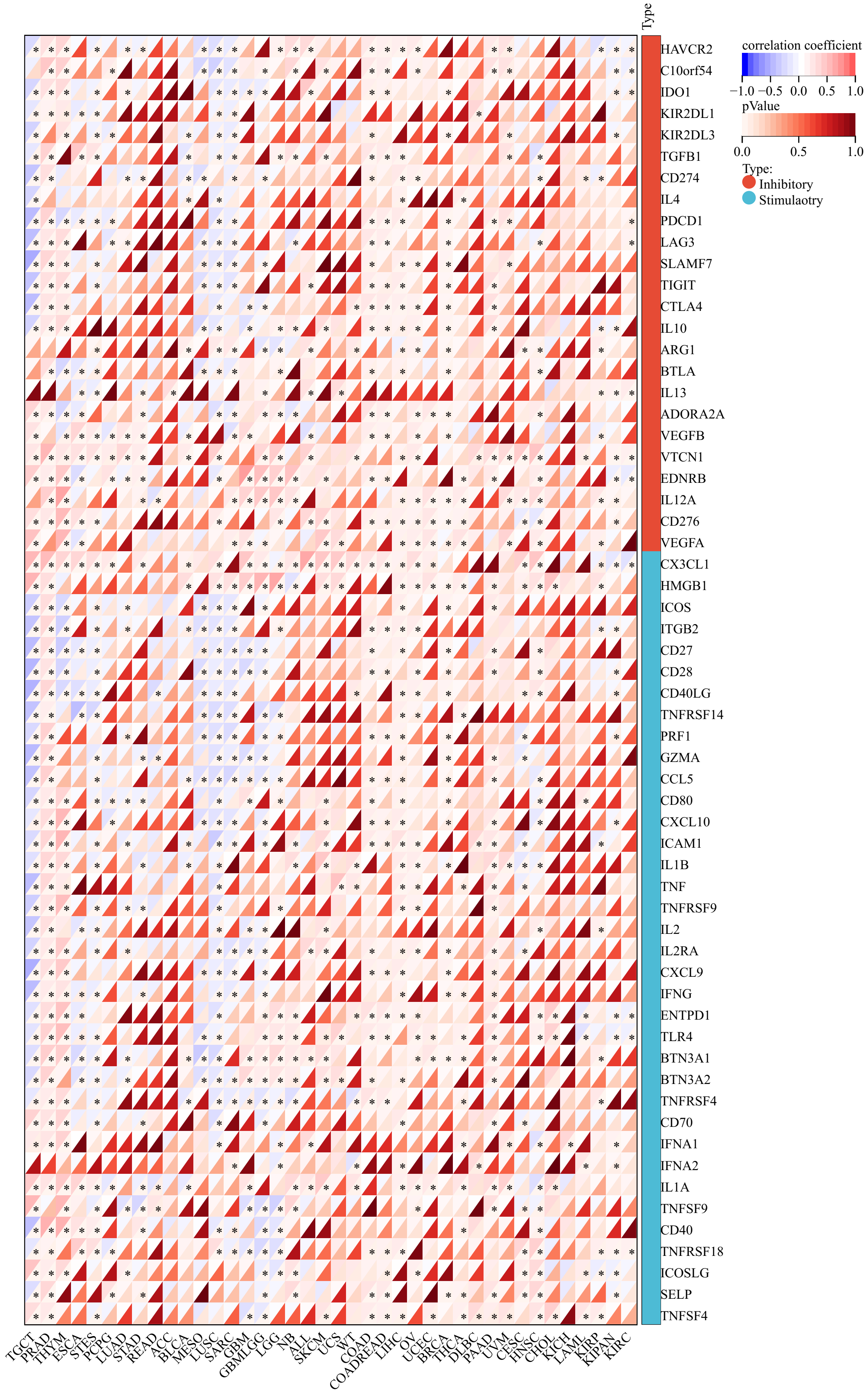

FigureS7:Immune checkpoint gene analysis (A):Immune checkpoint gene analysis of SOX2 in pan-cancer
